# Protein disorder–order interplay to guide the growth of hierarchical mineralized structures

**DOI:** 10.1038/s41467-018-04319-0

**Published:** 2018-06-01

**Authors:** Sherif Elsharkawy, Maisoon Al-Jawad, Maria F. Pantano, Esther Tejeda-Montes, Khushbu Mehta, Hasan Jamal, Shweta Agarwal, Kseniya Shuturminska, Alistair Rice, Nadezda V. Tarakina, Rory M. Wilson, Andy J. Bushby, Matilde Alonso, Jose C. Rodriguez-Cabello, Ettore Barbieri, Armando del Río Hernández, Molly M. Stevens, Nicola M. Pugno, Paul Anderson, Alvaro Mata

**Affiliations:** 1 Institute of Bioengineering, Queen Mary University of London, London, E1 4NS UK; 2 School of Engineering and Materials Science, Queen Mary University of London, London, E1 4NS UK; 3 Institute of Dentistry, Barts and The London School of Medicine and Dentistry, Queen Mary University of London, London, E1 4NS UK; 4 Materials Research Institute, Queen Mary University of London, London, E1 4NS UK; 5 Laboratory of Bio-Inspired and Graphene Nanomechanics, Department of Civil, Environmental and Mechanical Engineering, University of Trento, 38123 Trento, Italy; 6 Department of Materials, Imperial College London, London, SW7 2AZ UK; 7 Department of Bioengineering, Imperial College London, London, SW7 2AZ UK; 8 Institute of Biomedical Engineering, Imperial College London, London, SW7 2AZ UK; 9 G.I.R. Bioforge, University of Valladolid, CIBER-BBN, Valladolid, 47011 Spain; 10 Department of Mathematical Science and Advanced Technology, Japan Agency for Marine-Earth Science and Technology, Yokohama Institute for Earth Sciences, 3173-25, Showa-machi, Kanazawa-ku, Yokohama-city, Kanagawa 236-0001 Japan; 11 Ket-Lab, Edoardo Amaldi Foundation, Italian Space Agency, Via del Politecnico snc, 00133 Rome, Italy

## Abstract

A major goal in materials science is to develop bioinspired functional materials based on the precise control of molecular building blocks across length scales. Here we report a protein-mediated mineralization process that takes advantage of disorder–order interplay using elastin-like recombinamers to program organic–inorganic interactions into hierarchically ordered mineralized structures. The materials comprise elongated apatite nanocrystals that are aligned and organized into microscopic prisms, which grow together into spherulite-like structures hundreds of micrometers in diameter that come together to fill macroscopic areas. The structures can be grown over large uneven surfaces and native tissues as acid-resistant membranes or coatings with tuneable hierarchy, stiffness, and hardness. Our study represents a potential strategy for complex materials design that may open opportunities for hard tissue repair and provide insights into the role of molecular disorder in human physiology and pathology.

## Abstract (web summary)

There is evidence that disordered proteins play a role in the mineralization process. Here, the authors report on the development of elastin-like recombinant protein membranes using disordered-ordered interplay to investigate and guide mineralization.

## Introduction

Nature is rich with examples of sophisticated materials displaying outstanding properties that emerge from their specific hierarchical structure^1^. Materials such as nacre, bone, and dental enamel possess distinct structural organization at different length scales, which enhance their bulk material properties and functionality^2^. The capacity to create synthetic materials that emulate such ingenious architectures represents a major goal in materials science and an opportunity to tune and profoundly improve functionality^3^. In particular, the field of biomaterials would greatly benefit from the functionalities that can emerge from well-defined hierarchical organizations^4^.

Biomineralization, the process by which minerals are formed by living organisms under strict biological control, is responsible for the well-defined structure and subsequent function of mineralized tissues^5^. This process is based on a highly dynamic environment regulated by an organic matrix that nucleates and directs the hierarchical growth and morphogenesis of mineralized tissue^5^. The charge^6^, conformation^7^, supramolecular assembly^8^, and posttranslational cross-linking^9^ of specific macromolecules of the organic matrix have key multifunctional roles during the biomineralization process. For example, negatively charged domains in non-collagenous^10^ and non-amelogenin^7^ proteins are known to stabilize crystal nucleation, whereas the degree of collagen cross-linking in bone is known to affect its mineral density, microarchitecture, and stiffness^11^.

Tissues such as bone and nacre have motivated the development of synthetic mineralizing materials^12^. For example, several research groups have investigated ways to mineralize collagen intrafibrillarly, in order to mimic the natural mineralization process of bone tissue^13^. Others have reported materials that resemble the hierarchical structure and chemical composition of nacre using a β-chitin matrix^14^ and layer-by-layer polyelectrolyte-clay dispersions^15^. A particularly inspiring challenge has been, and continues to be, the pursuit of approaches that can recreate the distinctive apatite composition, hierarchical architecture, and corresponding properties of enamel^16^. Towards this goal, Yamagishi et al.^17^ and Yin et al.^18^ have developed inorganic chemical methods to grow aligned enamel-like apatite nanocrystals on dental enamel. However, approaches based on organic matrices offer the possibility to guide mineralization through a more biomimetic route based on tuneable organic–inorganic interactions^19^. Pioneering work by Moradian-Oldak and co-workers^20^ using amelogenin and Kniep and co-workers^21^ using gelatin has enabled the growth of aligned apatite nanocrystals directly on enamel surface. Nonetheless, the development of organized apatite nanocrystals with the distinctive hierarchical order of enamel expanding from the crystallographic-, nano-, micro-, and macro-scale, is still an exciting, yet unattained, goal^20^.

There is growing evidence that intrinsically disordered proteins (IDPs)  play a fundamental role in mineralization^22^. IDPs contribute in intermolecular interactions at the protein–mineral interface^23^. For example, Beniash et al.^24^ reported that amelogenin, a highly conserved IDP, undergoes a conformational change from disordered random coils into ordered β-sheet structures, upon interaction with the developing enamel crystals. This conformational change is known to guide crystal growth in enamel formation^25^. Recently, Carneiro et al.^26^ demonstrated that the distinctive hierarchical structure of mature enamel may require further conformational organization of amelogenin into amyloid-like nanoribbons. Synthetic mineralization platforms that can emulate features of these dynamic supramolecular matrices, exhibiting disorder–order optimization, may lead to the design of materials capable of recreating the structure and properties of biomineralized tissues^12,22^.

Elastin-like recombinamers (ELRs) are recombinant macromolecules based on the natural elastin recurrent motif Val-Pro-Gly-*X*-Gly (VPGXG), where *X* can be any amino acid apart from proline^27,28^. These biopolymers have generated great interest due to their biocompatibility, biodegradability, and capacity to be synthesized with a high level of molecular control^27^. In addition, ELRs can also serve as models of IDPs^29^, where their degree of disorder and order could be controlled to design supramolecular matrices that generate new functionalities^30,31^ and potentially guide mineralization. Recently, we have reported that intrinsically disordered ELRs can stabilize a precursor single crystal phase (brushite), which template the growth of a polycrystalline phase (apatite)^32^. A similar behavior is exhibited by other IDPs in biomineralization^33^.

Here we report the discovery and development of a process that exploits disorder–order interplay of ELRs to guide mineralization with remarkable control and hierarchy. The process enables the fabrication and tuneability of crystallographically aligned apatite nanocrystals. Those nanocrystals are organized into microscopic prisms, which grow together into well-defined macroscopic structures that can populate large volumes. The material exhibits high stiffness, hardness, and acid resistance, and can be fabricated as fully mineralized membranes or coatings over uneven surfaces including native tissues.

## Results

### ELR molecules and self-assembly

**Fig. 1:**
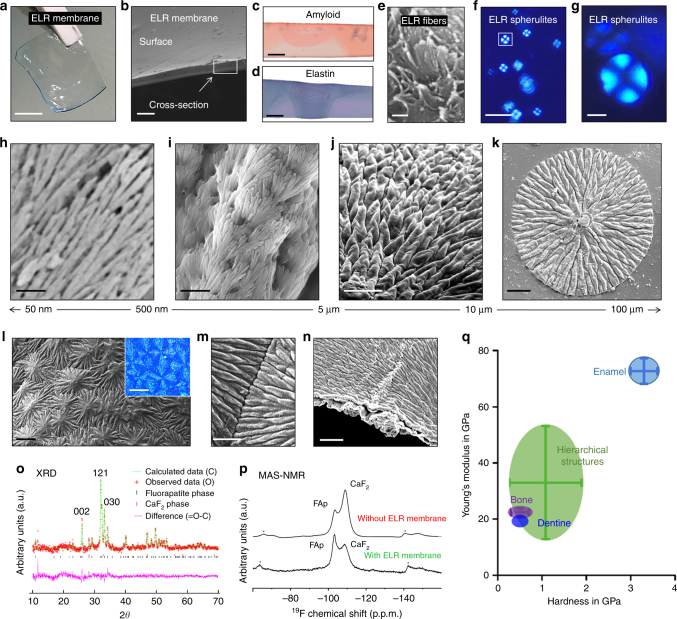
ELR spherulites and hierarchical mineralized structures. **a** Photograph of a transparent, robust, and flexible statherin-ELR membrane before mineralization. The membrane’s cross-section before mineralization **b** imaged by SEM and **c** histologically stained displaying a positive staining for both β-amyloid fibers and **d** elastin fibers with Congo Red and Elastin von Gieson stains, respectively. **e** SEM image of the membrane’s cross-section comprising of ELR nanofibers. **f**, **g** Polarized light microscope (PLM) images depicting the presence of ELR spherulites with the characteristic birefringent Maltese-cross pattern on the surface and within the bulk of the membranes. **h-k** SEM images of the top of an RGDS-ELR membrane after mineralization showing the hierarchical organization of the mineralized structures including **h** aligned fluorapatite nanocrystals that are **i, j** grouped into prism-like microstructures that further grow into **k** macroscopic circular structures. **l, m** The hierarchical structures grow until they meet each other, and **n** can mineralize completely thin membranes. **o** Rietveld refinement of an XRD pattern of mineralized membranes showing the fluorapatite nature of the crystalline phase with the typical Bragg peaks of apatite. **p** ^19^F solid-sate MAS-NMR spectra confirming the presence of fluorapatite and CaF_2_ (fluorite) phase at – 103 and – 108 p.p.m., respectively, with increasing fluorapatite peak intensity on the mineralized membrane (green) compared with those without the ELP membrane (red) at the same conditions. **q** Young’s modulus and hardness relationship between the mineralized structures and different mineralized tissues. Scale bars: **a** 5 mm; **b** 20 µm; **c**, **d** 40 µm; **e** 200 nm; **f** 10 µm; **g** 3 µm; **h** 200 nm; **i** 1 µm; **j** 10 µm; **k** 20 µm; **l** 30 µm; **l** (inset) 20 µm; **m** 5 µm; **n** 20 µm

Our process is inspired by the current understanding of dynamic organic matrices in biomineralization. We exploited recombinant technologies to design a supramolecular matrix that is based on a molecule that comprises both intrinsically disordered regions and negatively charged domains. The main molecule used is an ELR consisting of a main hydrophobic framework (VPGIG), a positively charged segment (VPGKG) with the amino acid lysine (K) for ELR cross-linking, and the highly acidic statherin-derived analog DDDEEKFLRRIGRFG (SN_A_15). Despite the fact that salivary statherin strongly binds to hydroxyapatite and inhibits mineral growth^34^, the SN_A_15^35^ segment comprising 15 amino acids situated at its N-terminal (DDDEEKFLRRIGRFG) is known to promote mineralization^36,37^. In addition, collagen membranes, ELR-coated glass, and membranes made from similar ELR molecules without the statherin-derived peptide or with the cell adhesive Arg-Gly-Asp-Ser (RGDS) were used as controls (Supplementary Table 1). When the ELR molecules are dissolved in anhydrous dimethylformamide (DMF) and dried at room temperature while cross-linked using hexamethyl diisocyanate (HDI), they self-assemble into a dense network of β-amyloid-like fibrils (Fig. 1a–e) and homogenously distributed three-dimensional (3D) ELR spherulites (Fig. 1f–g). On the other hand, no spherulites were observed on the collagen membranes (Supplementary Figure 1). Therefore, these organic supramolecular structures formed independently of the type of ELR used. This result suggests that the formation of spherulites is not dependent on the bioactive sequences nor the molecular weight of the ELRs, but on their VPGIG and VPGKG tropoelastin motifs.

### Protein-mediated mineralization process

Upon incubation in a solution supersaturated with respect to fluorapatite at physiological conditions and ionic concentrations, a mineralization process developed within the bulk of the ELR membranes (Supplementary Figure 2). This process resulted in the growth of distinctive hierarchically ordered mineralized structures (Fig. 1h–k) on both sides of the membranes (Supplementary Figure 3). These structures appeared to emanate from the ELR spherulites within the membrane independently of the ELR used. However, a higher number of mineralized structures were observed on membranes made from the statherin-derived ELR, suggesting that nucleation is enhanced with the quantity of acidic amino acids present within the ELR molecule^35–38^ (Supplementary Figure 4). In addition, the structures were not observed on the collagen membrane controls, whereas the ELR-coated glass surfaces only comprised flat platelet-like crystals (Supplementary Figure 5).

### Surface physical and chemical characterization

The mineralized structures exhibit a spherulite-like morphology with distinctive hierarchical architecture (Fig. 1k–l). At the crystallographic length scale, the material is apatitic in the form of elongated nanocrystals of on average 85.0 ± 22.0 nm thick. At the microscale, these crystals are organized further into prisms with an average diameter of 3.8 ± 0.9 µm and tens of micrometers in length. These microstructures grow radially into circular structures that can reach up to tens of microns in height and 1 mm in diameter (Supplementary Figure 6), while coming together, interlocking (Fig. 1m), and populating large uneven areas. This hierarchical mineralization process can produce membranes that are fully mineralized not only on the surface but also throughout their cross-section (Fig. 1n).

Rietveld refinement of X-ray diffraction (XRD) data demonstrated that the crystalline phase of the structures matches fluorapatite with a space group, unit cell size, and structural parameters matching fluorapatite values, as reported in the literature (Fig. 1o and Supplementary Note 1). This crystalline phase was confirmed by ^19^F magic angle spin-nuclear magnetic resonance (^19^F MAS-NMR) spectra^39^, which verified the presence of a fluorapatite peak at − 103 p.p.m. and a fluorite (CaF_2_) peak at − 108 p.p.m. (Fig. 1p). The presence of the organic ELR matrix seemed to promote the formation of fluorapatite phase at the expense of the undesirable fluorite phase, which is beneficial for potential biomedical applications^39^. Furthermore, Fourier-transform infrared (FTIR) spectroscopy analysis revealed spectra that exhibited non-stoichiometric apatite peaks after mineralization in co-existence with the amide peaks (corresponding to the ELR material) (Supplementary Figure 7). In addition, energy dispersive X-ray (EDX) spectroscopy point and mapping spectra also indicated the presence of calcium, phosphorus, and fluoride (Supplementary Figure 7) with atomic ratios similar to apatite crystals and dental hard tissues^40^. Furthermore, the process enabled the formation of similar hierarchical structures but without the use of fluoride (Supplementary Figure 8).

To demonstrate the functionality of the hierarchical structures, we characterized the mechanical properties by nanoindentation tests (Fig. 1q). As described in the section 'Tuneability of the process', we also demonstrate the possibility to tune the organization of the hierarchical stuctures in order to generate a range of physical properties, which can be used to target specific applications. The structures exhibited a Young’s modulus (*E*) of up to 33.0 ± 20.1 GPa and hardness (*H*) of up to 1.1 ± 0.8 GPa. The hierarchical structures’ *E* and *H* are higher than values reported for bone (*E* = 22.5 ± 1.3 GPa, *H* = 0.5 ± 0.2 GPa)^41^ and dentin (*E* = 19.4 ± 1.7 GPa, *H* = 0.5 ± 0.02 GPa)^42^ and nearly half of the values reported for dental enamel (*E* = 72.7 ± 4.5 GPa, *H* = 3.3 ± 0.3 GPa)^16^. However, it is important to mention that *E* and *H* for dental enamel can vary between 50–120 GPa and 2.5–6 GPa, respectively, depending on the crystal orientation, area within the tissue tested, technique used, and storage conditions^43,44^.

### Bulk characterization

**Fig. 2:**
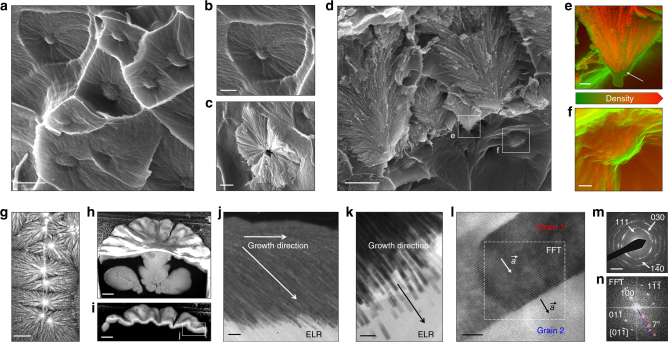
Bulk characterization. Organic–inorganic interactions within the bulk of the mineralized membranes. SEM observations revealed **a** the abundant presence of a dense pattern of spherulite-like structures with **b** a granulated central region at the bulk of the membranes’ cross-sections, which **c** template the growth of fluorapatite spherulites. **d** Near the membrane surface, mineralized structures with nanocrystals grow vertically toward the surface of the membrane. DDC-SEM images of **e** the core of fluorapatite spherulite structures located closer to the surface of the membrane and **f** deeper within the bulk of the membrane, revealing a thin low-density material (green) surrounding a denser (orange) one. **g** Backscattered electron (BSE) image showing brighter areas at the center of the structures, indicating the presence of mineral deep within the membrane. **h, i** FIB sectioning of the hierarchically mineralized structure resolving the deeper mineralized core structures located underneath the center of the structures. **j, k**TEM images from a FIB milling lift-out of a mineralized structure illustrating **j** the change in growth direction of the nanocrystals from parallel to the surface towards the bulk of the ELR membrane, and **k** the growing interface between the flat-ended inorganic crystals and the organic ELR material. **l** HRTEM image of a single fluorapatite crystal showing its growth orientation and crystal lattices. **m** SAED and **n** FFT analyses showing the characteristic diffraction pattern of the fluorapatite crystals (left) and its 10° co-alignment, respectively. Scale bars: **a** 2 µm; **b**, **c** 1 µm; **d** 2 µm; **e**, **f** 200 nm; **g** 20 µm; **h**, **i** 5 µm; **j** 200 nm; **k** 100 nm; **l** 10 nm; **m** 3 nm^−1^

Throughout the cross-section of the mineralized membranes, scanning electron microscopy (SEM) observations revealed the abundant presence of two types of structures. First, a dense pattern of spherulite-like structures with a granulated central region (Fig. 2a, b), which is likely associated with the observed birefringence. Some of these ELR spherulitic structures were mineralized and appeared to template the growth of fluorapatite spherulites made from nanocrystals similar to those observed on the hierarchical structures present on the surface of the membrane (Fig. 2c). Second, near the membrane surface, mineralized structures with nanocrystals were oriented at 78 ± 6° with respect to the membrane surface and appeared to grow vertically toward the surface of the membrane (Fig. 2d and Supplementary Figure 9). Interestingly, density-dependent color SEM (DDC-SEM), which simultaneously enables topographical and density assessment, revealed a thin low-density material (green) surrounding a denser (orange) one on both of these structures. According to the EDX spectral mapping, the less dense material was found to be rich in carbon, oxygen, and nitrogen, which are commonly found in organic materials. In contrast, the denser material exhibited an abundance of calcium and phosphorus, reflecting its inorganic nature and suggesting an intimate organic–inorganic interaction (Fig. 2e, f and Supplementary Figures 10-12).

SEM analysis using the backscattered electron mode (BSE) revealed that the centers of the hierarchical mineralized structures comprised mineralized cores deep within the bulk of the membrane (Fig. 2g, h, Supplementary Figure 13, and Supplementary Movies 1, 2). As the hierarchical structures spread radially on the surface, the nanocrystals in closer proximity to the ELR matrix changed their orientation gradually from parallel to the surface towards the inside of the membrane (Fig. 2 j). This behavior suggests a preference of the nanocrystals to grow within the ELR matrix. To further investigate this preferential growth and crystallographic orientation of the nanocrystals, ultrathin sections were milled via focused ion beam (FIB) and analyzed by high-resolution transmission electron microscopy (HRTEM) and selected area electron diffraction (SAED) (Fig. 2k–n and Supplementary Figure 14). The results demonstrate that the nanocrystals within the ELR matrix display a flat geometry at the end of their *c* axis (Supplementary Figure 15). In contrast, nanocrystals exhibiting a needle-like morphology have been associated with mineralization processes that do not rely on organic matrices to regulate their growth^18^. This offers further evidence that the ELR not only interacts at the basal plane of the fluorapatite crystals but also provides an optimum environment for their growth front within the matrix.

In addition, fast Fourier-transform analysis of the HRTEM images reveals that the nanocrystals are preferably oriented so that the *a* axis is perpendicular to the growth direction. Nanocrystals have a co-alignment of several degrees ranging between 7° and 10°, which may contribute to the spherulitic radial geometry of the hierarchical structures as seen in examples of other biomineralization systems^45^ (Fig. 2n and Supplementary Figure 16). These results demonstrate that ELR spherulites within the bulk of the membrane act as supramolecular organic frameworks that interact intimately with apatite crystals and template their growth into hierarchical mineralized structures.

### Tuneability of the process

**Fig. 3:**
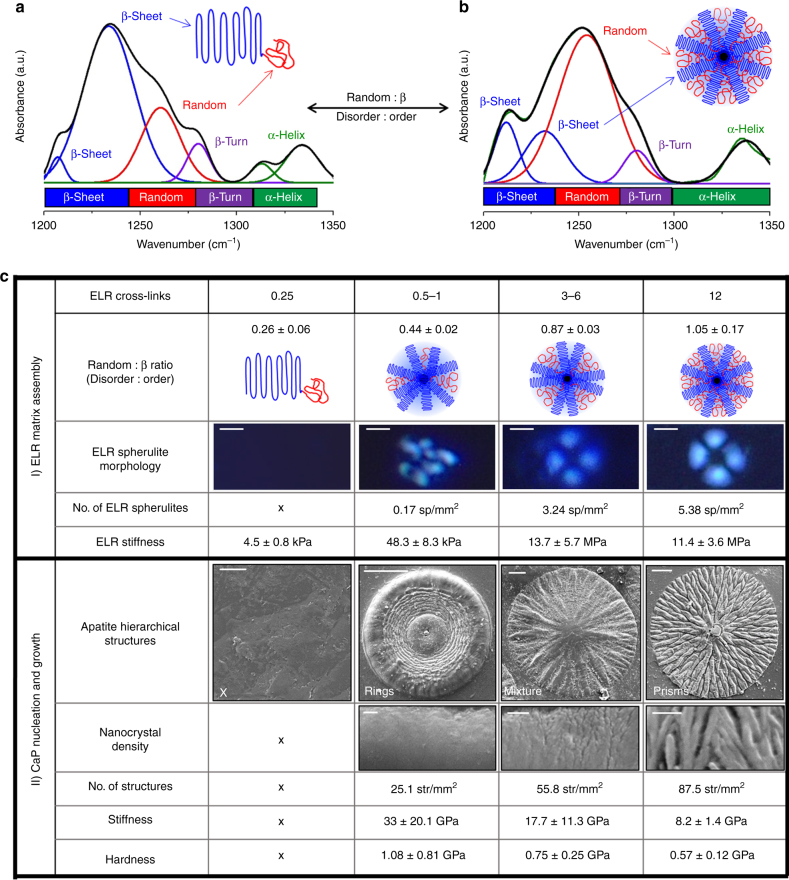
Protein disorder–order optimization and process tuneability. **a**, **b** Graphs showing the different levels of ELR order and disorder as a function of cross-linking. The levels of ELR ordered β-sheet structure and disordered random coil can be modulated and tuned, while maintaining β-turn and α-helix conformations nearly constant. **c** Table summarizing the tuneability of the process including during both ELR matrix assembly (Stage I) and the CaP nucleation and growth (Stage II). Scale bars: 3 µm (ELR spherulite morphology), 20 µm (apatite hierarchical structures), and 200 nm (nanocrystal density)

A major advantage of the process is the possibility to use and tune disorder–order interplay of the ELR molecules to design complex supramolecular organic environments that can control mineralization^22,46^. Through FTIR deconvolution of the amide III spectral region, a quantitative analysis of the protein secondary structure can be established^47^. We discovered that by systematically modifying the amount of cross-linker, it was possible to modulate the levels of ELR ordered β-sheet and disordered random coil conformation, while maintaining β-turn and α-helix nearly constant (Fig. 3a, b, Supplementary Figure 17, and Supplementary Table 2). In other words, tuning the amount of ELR cross-links during solvent evaporation enabled controlled access to different ELR disorder–order ratios within the resulting membrane. Using this drying/cross-linking approach, we found that it was possible to control the formation of ELR spherulites. When the ratio of disordered (random coil) to ordered (β-sheet) secondary structure (random: β ratio) was kept below 0.26 ± 0.06, no spherulites were formed. However, by gradually increasing the level of random coil (random : β ratios of 0.44 ± 0.02, 0.87 ± 0.03, and 1.05 ± 0.17), we were able to control the formation and number of ELR spherulites (0.17, 3.24, and up to 5.38 spherulites/mm^2^, respectively) (Fig. 3c(I)), demonstrating that ELR disorder–order optimization can be used to generate organic matrices with supramolecular control.

By modulating the ELR conformation in this manner, it appeared to be possible to tune the structural hierarchy of the mineralized structures and consequently the properties of the resulting material. As the supramolecular organization of the ELR matrix transitioned from a random : β ratio of 0.26 ± 0.06 to 1.05 ± 0.17, the microscale geometry of the hierarchical mineralized structures shifted from concentric rings to radial prisms (Fig. 3c (II)). However, independently of the microscale geometry, all structures comprised aligned nanocrystals but they tended to be denser in softer membranes (made with lower amount of cross-linker) compared with stiffer ones (made with higher amount of cross-linker). This control of the nanocrystal density enabled the possibility to tune both the Young’s modulus (*E*) and hardness (*H*) of the mineralized structures. Structures grown within softer membranes exhibited higher *E* (33.0 ± 20.1 GPa) and *H* (1.1 ± 0.8 GPa) compared with structures grown within moderate (*E* = 17.7 ± 11.3 GPa, *H* = 0.8 ± 0.3 GPa) and stiff (*E* = 8.2 ± 1.4 GPa and *H* = 0.6 ± 0.1 GPa) membranes. This increase in mechanical properties of the hierarchical mineralized structures on softer ELR membranes may result from the nanocrystals more easily displacing the ELR matrix and growing in closer proximity to each other. However, independently of the microstructure generated, the chemical composition remained the same (Supplementary Figure 18). These results demonstrate the capacity to program the hierarchical structure and resulting properties of the mineralized material by modulating disorder–order levels of the ELR organic matrix. We speculate that this possibility is in agreement with the increasingly recognized importance of IDPs in biomineralization^22^. Furthermore, these findings may provide insights into the role of IDPs in pathological mechanisms such as diseases of the central nervous system^48^ or cardiovascular calcification^49^.

The process can also be tuned by modulating other processing parameters. For example, increasing the number of negatively charged acidic domains within the ELR molecule/spherulite, such as those found in statherin-ELR, led to a higher number of mineralized structures. Also, controlling the kinetics of ionic consumption by maintaining a constant pH during the mineralization process considerably increased the size of the hierarchical structures up to a millimeter in diameter. Nonetheless, is important to highlight that hierarchical structures can also be formed in physiological ionic concentrations (2.5 mM Ca^2+^, 1.5 mM PO_4_^3−^) (Supplementary Figure 19).

### Mechanism of formation

**Fig. 4:**
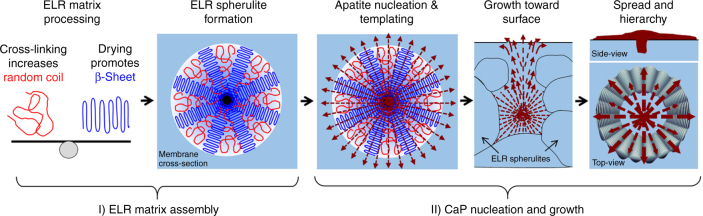
Mechanism of formation. A schematic illustrating the two stages of the proposed mechanism divided in (I) ELR matrix assembly and (II) calcium phosphate (CaP) nucleation and growth

Based on our data, we propose the following mechanism of (I) ELR matrix assembly and (II) calcium phosphate (CaP) nucleation and growth of the mineralized structures (Fig. 4).

### ELR matrix assembly

The protein-mediated mineralization process relies on the hydrophobic (VPGIG)/hydrophilic (VPGKG) block structure and disordered nature of the ELR molecules. In this first stage, during cross-linking, the ELR molecules organize into β-amyloid-like fibers, as previously predicted^50^, and spherulitic structures with the characteristic Maltese-cross pattern upon drying. As the DMF solvent evaporates, the ELR self-assembles transitioning from being random (disordered) in the DMF solution^51^ into a β-sheet conformation (ordered) in dry state. Although drying is known to induce molecular order^52^ (β-sheet conformation in our case), cross-linking prevents it by decreasing the molecule’s entropy, as evidenced by progressively inducing random coil by gradually increasing levels of cross-linking. In this way, the organic matrix can be easily modulated to systematically access different disorder–order compositions as well as different organizations of ELR spherulites. This is a critical capability of our process because, although the specific mechanism for the formation of protein-based spherulites remains vague^53^, it is known that these structures grow by triggering radially oriented crystalline regions located between amorphous chains^54^. Our approach enables the formation and modulation of these disordered-ordered regions. Furthermore, although proteins are known to form spherulites^55^, to our knowledge, this is the first time that a recombinant elastin protein has been reported to form such supramolecular structures.

### CaP nucleation and growth of mineralized structures

The 3D ELR spherulites within the matrix (membrane) then act as nucleating and templating sites for mineralization as CaP ions from the mineralization solution diffuse through the membrane. Membranes with a higher number of ELR spherulites comprised more mineralized structures (87.5 structures/mm^2^), whereas those with a lower number of ELR spherulites comprised less mineralized structures (25.1 structures/mm^2^). After nucleation, we propose that the rich ELR spherulites begin to template the growth of apatite nanocrystals and evolve into mineralized spherulites. This templating may be facilitated by the structural disorder of the ELR matrix, enabling both random and β structures, reported previously^22,26^.

ELR spherulites are formed within the cross-section of the ELR matrix and, like polymeric spherulites, grow until they meet adjacent ones. The spherulites located closer to the surface of the membrane have fewer limitations to grow toward and on the surface of the membrane. In this way, mineralized structures templated by ELR spherulites closer to the surface of the membrane not only have open space to grow but also more access to CaP ions. Once on the surface, the mineralizing structures continue to spread until they meet others. Despite changes in the microscale architecture of mineralized structures growing on membranes with different amounts of cross-linking, the outward alignment of the nanocrystals remains the same, suggesting that the microscale organization is at least in part dependent on the properties of the matrix. To explore this, we used a model based on the wrinkling of a thin circular annulus (represented by the hierarchical mineralized structure) supported by elastic foundations (represented by the underlying ELR matrix)^56^. The bending stiffness of the mineralized structures in the principal direction becomes negligible and would respond with a circumferential out-of-plane deflection (the wrinkles) when radial compressive loads (as a result of the radially outward nanocrystal growth) are applied to either the inner or outer edge. In this scenario, the model predicts that softer ELR membranes would generate wrinkles with circular symmetry (the ring morphology), while stiffer ELR membranes would generate wrinkles with radial symmetry (i.e., prismatic morphology) (Supplementary Figure 20). It is also important to mention that differences in ionic diffusion across the membrane as a result of the various conformations of the ELRs may also have a role in the morphology of the mineralized structures (Supplementary Table 3)^57^.

Our approach takes advantage of the disordered nature of ELR molecules to trigger a supramolecularly organized organic framework capable of controllably templating the growth of apatite crystals at multiple length scales. This mechanism goes beyond biomimicry and opens up the possibility to not only modulate mineralization but also to explore ways of utilizing disorder–order interplay for the generation of functional materials.

### Application in dental tissue repair

**Fig. 5:**
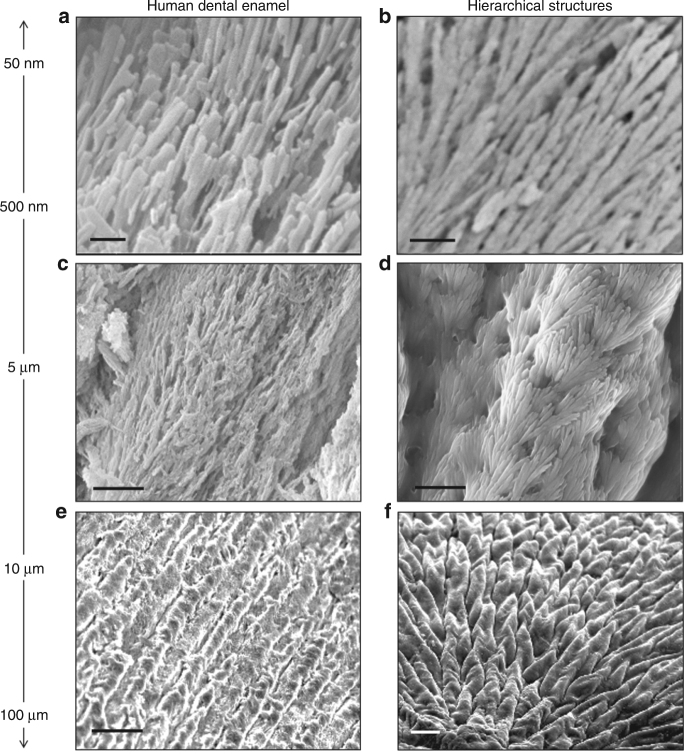
Resemblance between enamel and the hierarchical structures. SEM images illustrating the close resemblance of human dental enamel **a** to the hierarchically ordered mineralized structures **b** grown on a RGDS-ELR membrane at multiple length scales. Scale bars: **a**, **b** 200 nm; **c**, **d** 1 µm; and **e**, **f** 20 µm

Hierarchical structures have a key role in mineralized tissues such as bone, dentin, and enamel. In particular, dental enamel offers a unique structure/function relation (Fig. 5a, c, e), which has not yet been recreated^16^. Given, the vast clinical need and potential impact of engineering more efficient materials to replace lost/diseased enamel, we conducted in vitro proof-of-concept studies to investigate the potential use of the hierarchical mineralized structures (Fig. 5b, d, f) for dentin hypersensitivity as a mineralizing bandage to occlude exposed dentinal tubules.

**Fig. 6:**
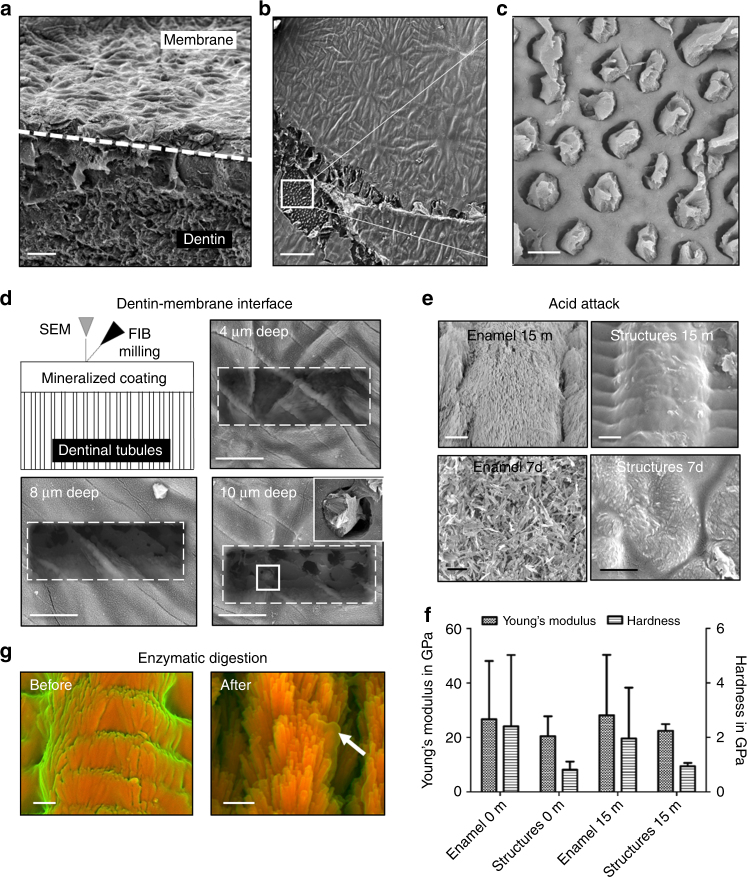
Dental applications of the hierarchical structures. **a** Application of the in-situ cross-linked ELR membrane conformed over the rough and uneven surface of exposed human dentin, exhibiting the hierarchical mineralized structures as a coating on top of the native tissue. SEM images depicting **b** a removed section (white square) of the mineralized membrane with **c** the nanocrystals infiltrating, binding, and occluding the open dentinal tubule structures. **d** FIB milling of the mineralized coating at different depths to observe the dentin-membrane interface, which exhibits infiltration of nanocrystals occluding the dentinal tubules. **e** SEM images revealing the effect of the acid attack at different time-points (15 min, and 7 days) on both human dental enamel and the hierarchical mineralized structures. **f** Graph illustrating the Young’s modulus and hardness of the human enamel and mineralized structures after the acid attack. **g** DDC-SEM images of the hierarchical mineralized structures after the enzymatic digestion, showing a partially remaining organic material (arrow). Scale bars: **a**, **b** 50 µm; **c** 3 µm; **d** 10 µm; **e** 1 µm; **g** 500 nm. Error bars are represented as SD, *n* = 10

Membranes were fabricated directly on both etched and rough surfaces of human dentin and mineralized for 8 days. SEM observations confirmed that the hierarchically mineralized membranes grew, adhered, and conformed to the surface of the etched dental tissues (Fig. 6a–c). Integration between the hierarchical structures and the dental tissues was observed at the dentin-membrane interface, where the nanocrystals infiltrated and blocked dentinal tubules (Fig. 6d). Furthermore, to assess the acid resistance of the mineralized coatings, we conducted acid attack experiments on both mineralized membranes and dental enamel. The mineralized structures exhibited comparable acid resistance to dental enamel after 15 min of exposure (Fig. 6e, f). It is probable that the acid resistance exhibited by the mineralized structures is related to their intrinsic fluorapatite crystalline phase in comparison to the carbonated hydroxyapatite phase found in enamel^58^. As expected, after 7 days of exposure to acid, the inorganic content of the mineralized membranes and dental enamel were disturbed. Interestingly, the stiffness of ELR membranes after the 7-day acid attack was similar to that of unmineralized membranes, suggesting that the ELR matrix was preserved after the attack (Supplementary Figure 21). This preservation could potentially enable a remineralization treatment once the acid attack subsides. However, confirmation of this hypothesis would require further experimentation, which is beyond the scope of the current study. Another potential challenge encountered by dental tissues is exposure to proteases in saliva^59^. Therefore, membranes were tested for enzymatic degradation by elastase exposure. DDC-SEM observations confirmed the presence of the nanocrystals (high-density material) and a reduction of the low-density material (organic matrix) (Fig. 6g). This result demonstrates that the prismatic structures can maintain their organization in spite of the reduction of the organic matrix around them. Great progress has been made aiming to repair enamel using either wet chemical processes^18^ or organic matrices^20,21^. Our process provides a potential route to control the growth of fluorapatite crystals with tuneable hierarchical order and mechanical properties.

## Discussion

This work demonstrates the possibility to use IDPs to modulate disorder–order ratio to program mineralization. The process is based on a tuneable supramolecular organic matrix that enables the growth of aligned nanocrystals into hierarchical mineralized structures. These materials exhibit high stiffness, hardness, and acid resistance and can be fabricated as fully mineralized membranes or coatings over uneven surfaces including native tissues. In addition, the simplicity and versatility of the mineralization platform opens up opportunities to tackle regenerative challenges of mineralized tissues. Furthermore, the work not only introduces an exciting strategy to design and grow materials, but also provides insight into phenomena emerging from disorder–order optimization. As such, our study is highly relevant for different fields including, e.g., materials science, biomineralization, and structural biology.

## Methods

### Membrane fabrication and ELR glass coating

Membranes were fabricated by dissolving different ELR molecules as shown in (Supplementary Table 1) in anhydrous DMF at room temperature in a low-humidity conditions (< 20%) inside a polymer glove box. Then the resultant solution was cross-linked using HDI, drop-casted on top of  polydimethylsiloxane (PDMS) substrate, left to dry overnight, and then washed with deionized water for 3 days and stored at 4 °C. Systematic processing variations in order to control the stiffness/ELR conformation of the membranes by changing the cross-linker to lysine ratios (0.25, 0.5, 1, 3, 6, and 12). Collagen membranes processed as above, were used as controls. Similar fabrication procedures were undertaken to fabricate membranes on the dentin substrates. For ELR glass coating, 100 µg of ELR was dissolved in deionized water and drop-casted onto the borosilicate glass substrates, and left to dry. After drying the membranes were observed under polarized light microscope with a cross-polarizer to check the formation of ELR spherulites.

### Enamel and dentin discs preparation

Extracted human non-carious teeth (with approval from Queen Mary Research Ethics Committee QMREC2008/57) were stored at 4 °C in deionized water refreshed every 7 days until needed. Each tooth was carefully mounted on a holder and placed inside the diamond cut-off machine (Accutom-5, Struers A/S, Ballerup, Denmark) by aid of a compound material, the required *X* and *Y* starting positions along with *Y* stop position were selected and saved. Teeth were cut across their axes into disks, the thickness of each dentin disc was about 500 µm. The tooth sections were carefully polished using a polishing unit (Kent 4, automatic lapping and polishing unit) by aid of silicon carbide (SiC) grinding papers (CarbiMet^TM^) from coarse to fine as follows (P600, P1000, P2500, and P4000). Subsequently, the samples were polished using polishing cloth and diamond suspension waterbase (Metprep^TM^) as follows (3, 1, and 0.25 µm). Finally, the dentin disks were acid etched using 6% citric acid for 2 min^60^; however, enamel samples (longitudinal sections) that were used as controls for SEM images were etched using 38% phosphoric acid for 30 s^61^. Then the ELR membranes were fabricated in situ on top of the dentin disks was conducted.

### Crystal growth experiment

Hydroxyapatite powder (2 mM) and 2 mM of sodium fluoride were added to 100 ml of deionized water with continuous stirring. Subsequently, 69% nitric acid was added dropwise into the solution slowly until the powder was completely dissolved. Ammonium hydroxide solution (30%) was added dropwise until the pH was readjusted to 6.0^62^, and then different ELR membranes were placed at the bottom of beaker and incubated for eight days at 37 °C using a temperature-controlled incubator (LTE Scientific, Oldham, UK). Furthermore, initial ionic strength was varied to mimic natural saliva at concentrations of 2.5 mM and 1.6 mM of calcium and phosphate, respectively. Quantification and analysis of the mineralized structures were conducted by ImageJ.

### SEM, DDC-SEM, and EDX spectroscopy

Samples were mounted after being dried on aluminum stubs via self-adhesive tape and were coated using an auto sputter coating machine with a conductive material. Samples were analyzed using an FEI Inspect F (Hillsboro, OR, USA). Their surface topography was observed using a secondary electron detector. A BSE detector was used to assess the variation in density within each sample. Furthermore, the elemental analysis was carried out using INCA software. Point and mapping spectra collection at areas of interest were carried out using an EDX detector (INCA x-act, Oxford Instruments) at an accelerating voltage of 10 kV. In other instances, samples were investigated using SEM (Gemini 1525 FEGSEM), operated at 10 kV. The instrument was equipped with both an inlens detector that recorded secondary electrons, and a backscatter electron detector. The DDC-SEM images were obtained by imaging the same region with both inlens mode and backscatter mode. Using ImageJ software, both images were stacked and the inlens image was assigned to the green channel whereas the backscatter image was assigned to the red channel^63^.

### Focused ion beam-SEM

FIB-SEM was undertaken using an FEI Quanta 3D ESEM (Hillsboro, OR, USA) for which the gallium ion beam parameters were set to 30 kV and 1 nA, in order to cut trenches from the mineralized structures. Areas to be milled on the samples were selectively protected from the gallium source by depositing 2 µm of platinum coating. For the 3D reconstructed data, mineralized structures were milled, where each slice was 73 µm wide, 65 µm tall, and 20 µm deep, while the images were captured at 5 kV with magnification of × 4000 after each cut using auto slice and view software (FEI) with resolution of 1024 × 884 were then reconstructed using ImageJ (National Institute of Health, USA) and Drishti (Australia National University, Canberra, Australia) softwares^64^. TEM specimens were prepared using a FEI Quanta 3D ESEM by focused Ga + ion beam milling. The wedge cut technique followed by an in situ welding lift-out and thinning was applied. Thinning using a 30 kV ion beam and currents down to 28 pA was done from both sides of the specimen at a 2° incidence angle until the lamella was about 150 nm thick. The final low-kV cleaning was performed at 16 kV, 28 pA, and 1° incidence angle.

### Histological characterization

ELR membranes were embedded in paraffin wax blocks, cut into sections of about 3 µm, and stained with Congo Red and Elastin Von Gieson to visualize under optical light microscope its amyloid and elastin content, respectively.

### Attenuated total reflection FTIR

FTIR analysis was conducted using the FTIR Spectrum GX (PerkinElmer®, Waltham, MA, USA). Membranes before and after mineralization were properly secured over the IR window before scanning equipped with an attenuated total reflection attachment. Human non-carious dental enamel powder (kindly supplied by Professor Colin Robinson, University of Leeds) was also analyzed for comparison purposes. The program was set to take the average of 128 scans at resolution of 4 cm^−1^ after subtracting the background, and were analyzed at a wavenumber of 4000 cm^−1^ to 450 cm^−1^ in respect to % of transmittance and % of absorbance for inorganic and organic samples respectively. Usually, amide I spectral region (1700–1600 cm^−1^), is most commonly used to detect the secondary structure of proteins because of its strong signal, while it has several limitations, including a strong interference from water vibrational band, relatively unstructured spectral contour, and overlap of revolved bands correspondingly to various secondary structures. In contrast, amide III that was investigated in our study, at spectral region (1350–1200 cm^−1^), even if relatively weak in signals, does not have the above limitations. Easily resolved and better defined amide III bands are suitable for quantitative analysis of the protein secondary structure. By using Origin software, amide III region has been successfully deconvoluted and used for determination of α-helix and β-sheets, bands corresponding to β-turns and random coils. The assignments of spectral bands were as follows: 1220–1250 cm^−1^ for β-sheets, 1250–1270 cm^−1^ for random coils, 1270–1295 cm^−1^ for β-turns, and 1295–1330 cm^−1^ for α-helix^47^.

### X-ray diffraction

Powder diffraction was conducted at room temperature to elucidate the phase composition of the mineralized membrane, using an X’Pert Pro X-ray diffractometer (PANalytical, B.V., Almelo, Netherlands) with flat plate *θ*/*θ* geometry and Ni-filtered Cu-Kα radiation at 45 kV and 40 mA, where Kα1 and Kα2 equal 1.540598 and 1.5444260 Å respectively. The 2*θ* range of the diffraction pattern was taken from 5° to 70° with a step size 0.0334° and data were collected continuously with an equivalent step time of 1600 s using a PANalytical X’Celerator solid-state real time multiple strip (RTMS) detector. Rietveld modeling was performed using X’pert highscore (3.0*e*) with the ICDD PDF-4 + database for 2014^65^.

### ^19^F magic angle spin-NMR

In order to investigate the fluoride interactions present in both the powder collected from base of the beakers with no ELR membranes (as a control) and the mineralized membranes, all samples were crushed into fine powder using pestle and mortar, and then analyzed. Solid-state ^19^F MAS-NMR analysis was conducted using a 14.1 Tesla spectrometer (600 MHz, Bruker, Coventry, UK) at a Larmor frequency of 564.5 MHz under spinning conditions of 22 kHz in a 2.5 mm rotor. The spectra were acquired from a single-pulse experiment of 60 s recycle duration, by using a fluorine free background probe. The ^19^F chemical shift scale was calibrated using the − 120 p.p.m. peak of 1 M of NaF solution along with trichloro-fluoro-methane (CFCl_3_), as a second reference. Spectra were acquired for 4 h with accumulation of 240 scans, whereas for the protein membranes there was an accumulation of four runs each for 4 h.

### Transmission electron microscopy

TEM microscopy was performed on the FIB-prepared lamellas using a JEOL JEM 2010 (JEOL Ltd., Tokyo, Japan) operated at 120 kV. The obtained images were analyzed using the Gatan Microscopy Suite^®^ (GMS 3) software. For the analysis of crystal phases present in the samples, *d*-values obtained from SAED patterns were compared with *d*-values obtained from the same samples using XRD measurements and the Powder diffraction file - PDF2 database (ICDD, USA, release 2009).

### Atomic force microscopy nanoindentation

Unmineralized samples were attached to a petri dish using a drop of cyanoacrylate adhesive, and left for a minute for the adhesive to dry. Samples were then immersed in distilled water. Young’s modulus measurements were taken with a JPK Nanowizard-1 (JPK Instruments, Germany) in force spectroscopy mode, mounted on an inverted optical microscope (IX-81; Olympus, Japan). Quadratic pyramidal cantilevers (MLCT; Bruker, MA, USA) with a spring constant of 0.07 N/m and half-angle to face of 17.5° were used for indentation. The sensitivity of cantilevers was determined before measurements by measuring the slope of the force-distance curve in the atomic force microscopy (AFM) software on an empty region of a petri dish. Indentation was carried out with an approach speed of 5 µm/s and a maximum set force of 1 nN. Measurements were taken multiple times per region and in multiple regions per sample. The Young’s modulus was calculated by fitting the contact region of the approach curve with the Hertz Contact model^66^ using the JPK software, using the above constants and calibrated cantilever sensitivity. Graphs were plotted with GraphPad Prism software, using a *P*-value of 0.05.

### Young’s modulus and hardness measurements

Before the measurements, the nanoindenter was calibrated against a reference sample that was fused silica in our case, as suggested by the machine supplier. Calibration was performed using the same method that was then implemented for tests on dental and membrane samples. Mineralized membrane samples were glued flat onto an aluminum holder. Enamel samples were put into a mold filled with fluid resin. After resin curing, enamel samples were polished in order to have a flat surface where to perform the mechanical tests. Nanoindentation tests were carried out through a nanoindenter (iNano) by Nanomechanics, Inc. (maximum indentation load of 50 mN), which was used in dynamic mode in order to monitor the variation of the young’s modulus and hardness as a function of the indentation depth. In order to reduce the influence of the soft underneath membrane on the mechanical properties of mineralized samples, we considered the mechanical properties measured at the outmost surface, which corresponds to an indentation depth of 30 nm.

### Ionic strength measurements

The concentration of calcium and fluoride ions in the mineralizing solution was analyzed using an ion‐selective electrode (ISE) in combination with a potassium nitrate reference electrode (ELIT Ion Analyser, Nico 2000 Ltd, Harrow, UK), four channel ion analyzer and computer interface software. The ISE was calibrated by standard solutions within the concentration range employed for both calcium and fluoride. A straight line of mV vs. concentration plotted to provide the calibration data.

### Acid attack experiments

Both human dental enamel and mineralized membranes were subjected to 0.1 M of acetic acid adjusted to pH 4.0 and incubated at 37 °C for different time points^67^.

### Enzymatic digestion

Mineralized membranes were subjected to elastase (from hog pancreas source) digestion^68^ after optimization of the concentration adjusted to 15 U/ml for 72 h at 37 °C.

### Swelling measurements

Swelling measurements to yield diffusion coefficient values at different cross-link densities. A circle was punched out of each ELR membrane (different cross-link densities) using a 0.5 cm biopsy punch. The dry weight and dimensions of each membrane were then recorded using a micro-balance and micrometer. Each membrane was immersed in a petri dish full of deionized water. The petri dish was placed under an optical microscope with a calibrated scale. The diameter and thickness of the membrane were measured as a function of time over the following time points: at 30 s intervals from 0 to 10 min, at 10 min intervals from 10 to 60 min, at 60 min intervals from 60 to 480 min, and at 24 h intervals between 480 and 2880 h. The moment the membrane was placed inside the petri dish was taken as *t* = 0. These measurements were carried out at room temperature. Analyses and calculations were conducted using ImageJ implementing Tanaka and Fillmore equations^69^.

### Data availability

The data that support the findings of this study are available from the authors on reasonable request, see author contributions for specific data sets.

## Electronic supplementary material

Supplementary Information

Description of Additional Supplementary Files

Supplementary Movie 1

Supplementary Movie 2
