# Supplementary material for: Protein disorder–order interplay to guide the growth of hierarchical mineralized structures
Source: Nat Commun. 2018 Jun 1;9:2145. doi: 10.1038/s41467-018-04319-0 (PMC5984621; doi:10.1038/s41467-018-04319-0)
Supplement: Supplementary file 1 — Supplementary Information [file 41467_2018_4319_MOESM1_ESM.pdf]

Supplementary Information

Protein disorder-order interplay to guide the growth of hierarchical mineralized structures

Elsharkawy et al.

Type of ELR	Sequence (bioactive sequence in red)	Isoelectric point (pI)	Molecular weight	Inverse transition temperature (in DW)	
				**pH**	**T_t (°C)**
Statherin-rich ELR	[[(VPGIG)$_2$(VPGKG)(VPGIG)$_2$]$_2$ DDDEEKFLRRIGRFG [(VPGIG)$_2$(VPGKG)(VPGIG)$_2$]$_2$]$_3$	9.9	31.9 kDa	3.5	>60
				7.2	23
				10.5	25
				**pH**	**T_t (°C)**
RGDS-rich ELR	[[(VPGIG)$_2$(VPGKG)(VPGIG)$_2$]$_2$AVTGRGDSPASS[(VPGIG)$_2$(VPGKG)(VPGIG)$_2$]$_2$]$_6$	11.1	60.6 kDa	3.5	39-41
				7.2	35-37
				10.5	26-28
				**pH**	**T_t (°C)**
ELR control	(VPGIG VPGIG VPGKG VPGIG VPGIG)$_{24}$	11	51.9 kDa	3.5	39-41
				7.2	34-36
				10.5	24-26

Supplementary Table 1. Table showing the different ELR molecules along with isoelectric points, bioactive sites, and molecular weights. Bioactive sequences are shown in red.

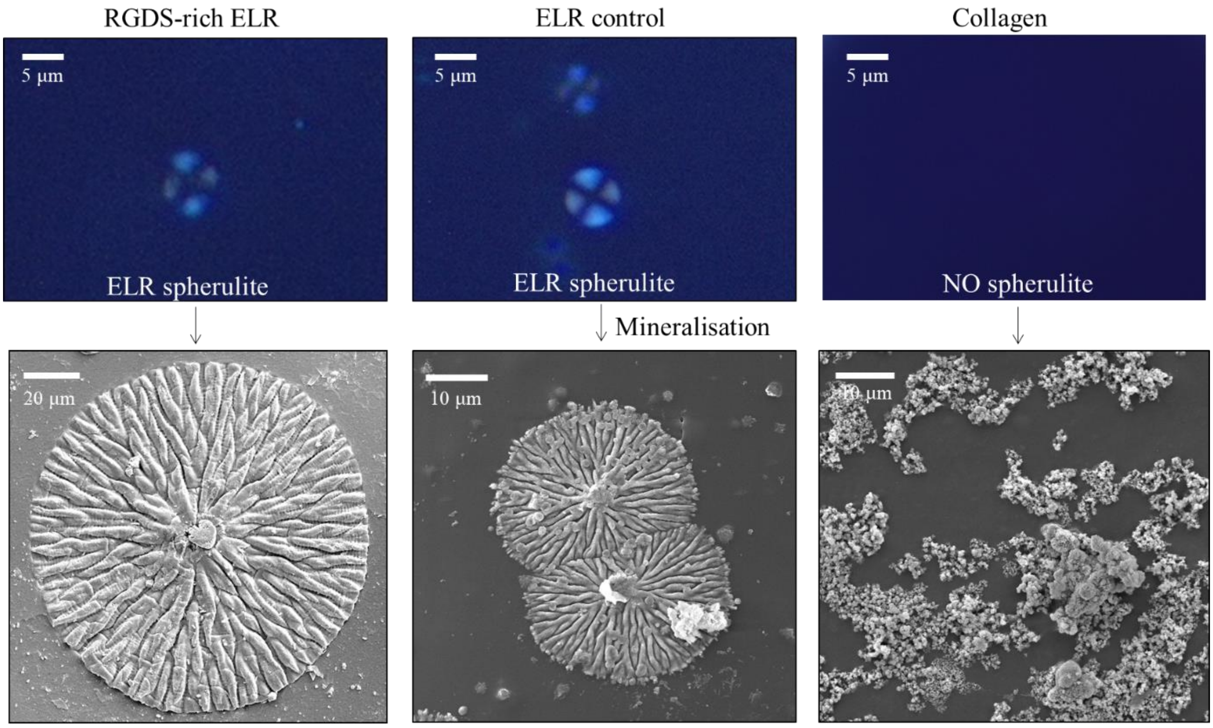

Supplementary Figure 1. ELR supramolecular spherulites formed independently of the type of ELR used, suggesting that the acquired conformations are not dependent on the bioactive sequences nor the molecular weight of the ELRs, but rather on the main VPGIG and VPGKG tropoelastin motifs. Furthermore, no spherulites were observed on the collagen membranes. These results demonstrate that the formation of the spherulites depends on the main ELR sequence. To further confirm, mineralization experiments were conducted, where the ELR spherulites evolved to mineralized spherulites.

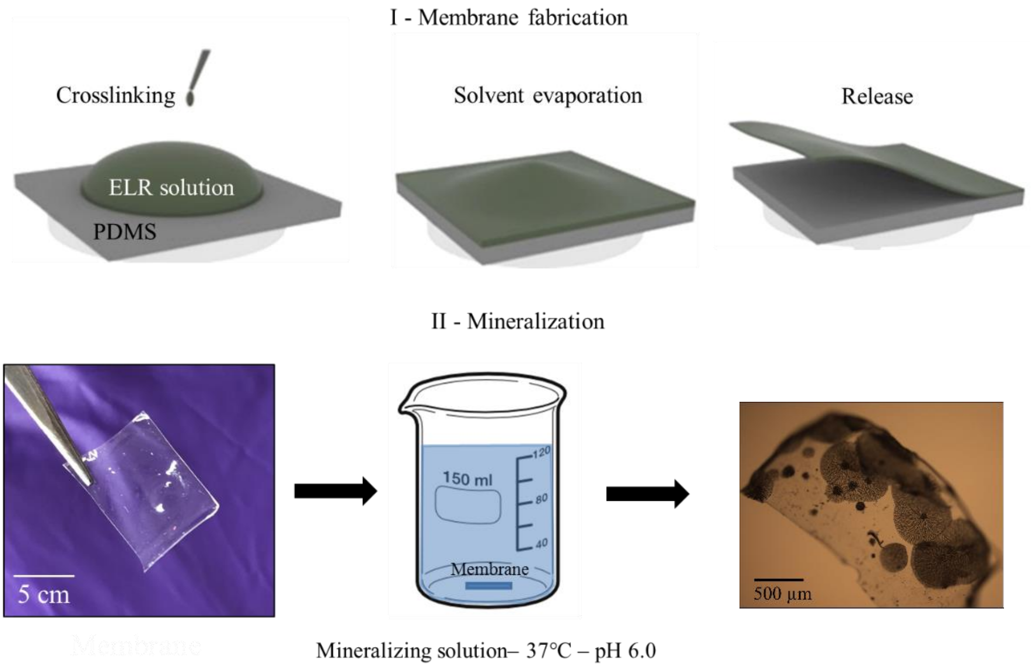

Supplementary Figure 2. Schematics illustrating the fabrication of the ELR membranes (top) and the mineralization process (bottom).

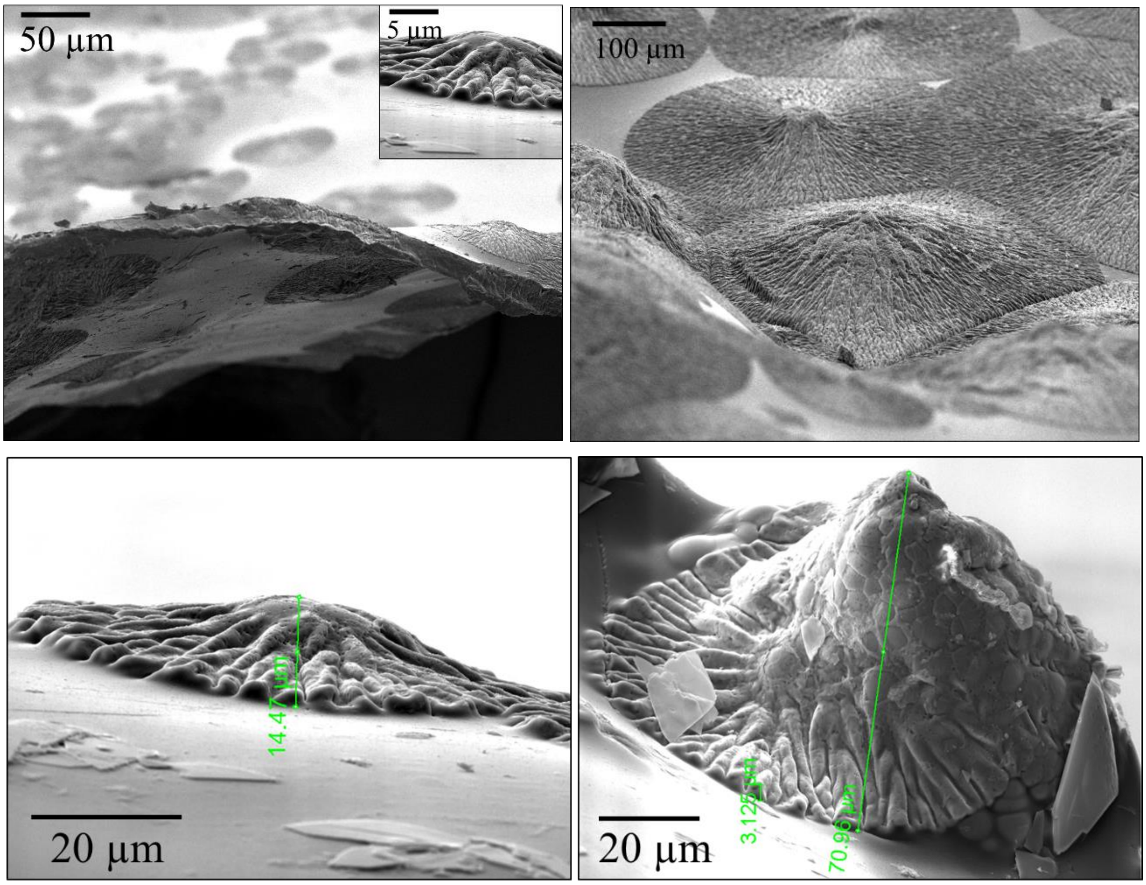

Supplementary Figure 3. SEM images showing the growth of the hierarchical mineralized structures on both sides of the RGDS-rich ELR membranes, as well as side views of the structures with different heights.

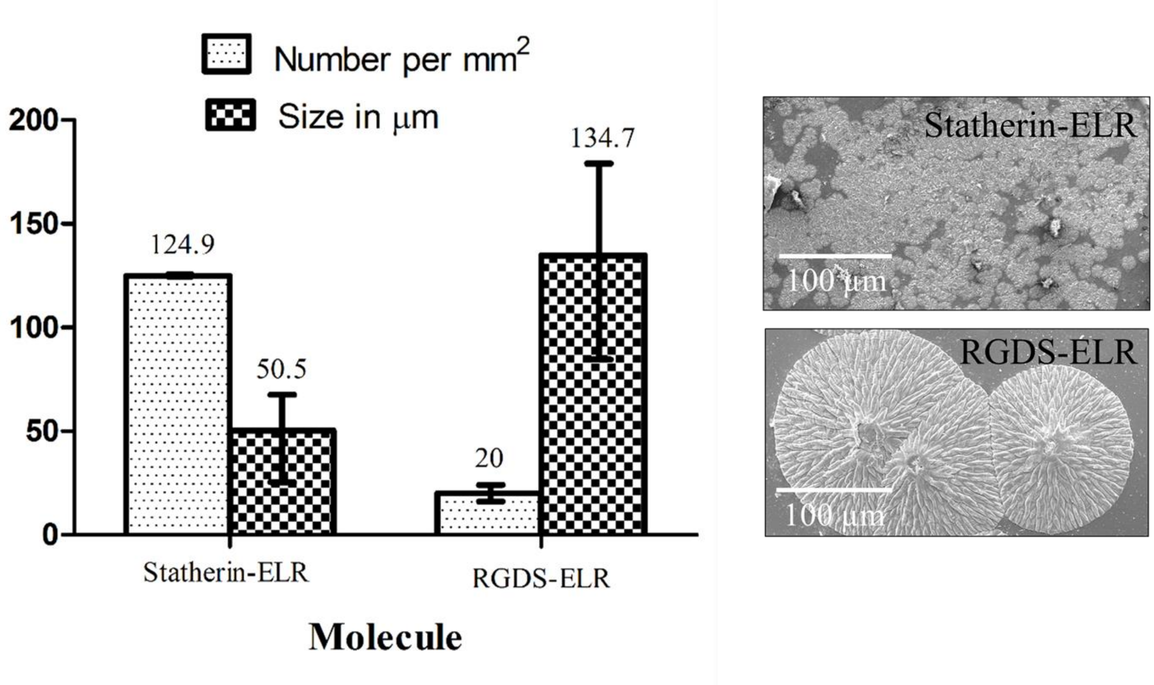

Supplementary Figure 4. Quantification of the number and size of the hierarchical mineralized structures of different molecules (statherin-ELR vs RGDS-ELR). An increase in the number of the structures can be observed on statherin-ELR compared to RGDS-ELR. On the other hand, the size of the mineralized structures is increased on the RGDS-ELR membranes compared to the statherin-ELR ones. This further evidences that the statherin-ELR membranes can promote more nucleation points compared to RDGS-ELR. Since the nucleation is enhanced on the statherin-ELR (as exhibited by the high quantity of the structures), the crystal growth is delayed (as exhibited by the smaller size of the structures). Errors bars is represented by standard deviation (SD) n = 3.

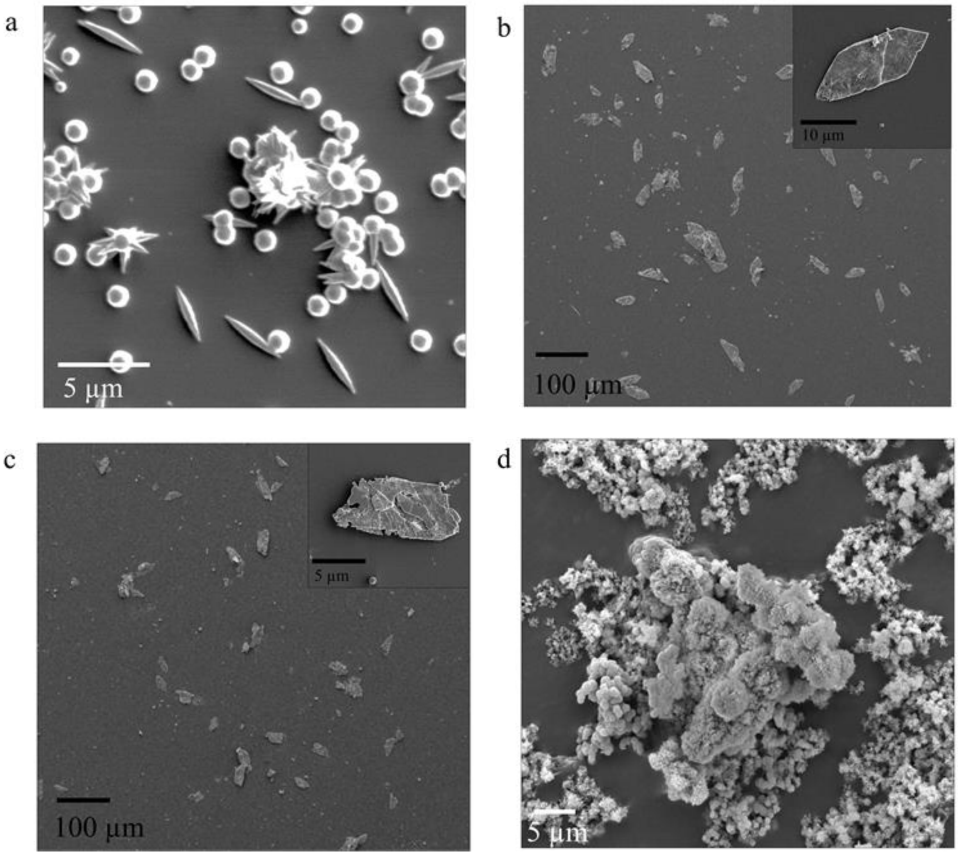

Supplementary Figure 5. SEM images of the crystal morphology on; a) uncoated borosilicate glass showing the characteristic needle-like and ball-like morphology; and borosilicate glass coated with ELRs b) statherin-ELR, and c) RGDS-rich ELR showing flat plate-like crystals. Similarly collagen membranes showed disordered crystal growth (d).

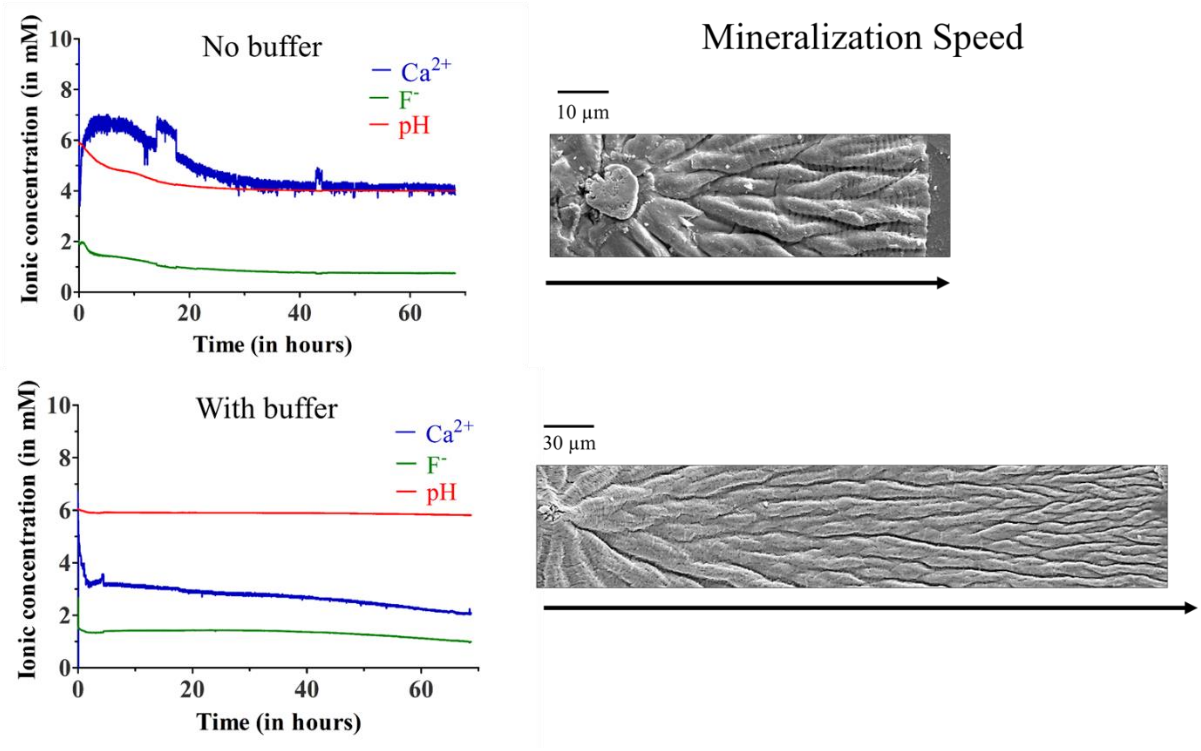

Supplementary Figure 6. Ion selective electrode (ISE) measurements and SEM images (right), showing the free ion concentration in the system as a function of time with (bottom) or without (top) the use of the BIS-TRIS buffer (top), that controls the pH of the system, the system reaches steady-state conditions earlier under constant pH, and hence more calcium consumption, faster mineralization, and larger mineralized structures (bottom) up to almost 1 mm in diameter.

Supplementary Note 1. After Rietveld refinement of XRD, the hexagonal unit cell parameters (space group $P6_3/m$ No. 176) are a= 9.3757(16) and c= 6.8841(12) Angstroms. The unit cell therefore has a volume of 524.1 (1) cubic Angstroms. This results further confirm that the crystalline phase of the structures is fluorapatite with a space group, unit cell size and structural parameters matching fluorapatite values, as reported in the literature[1].

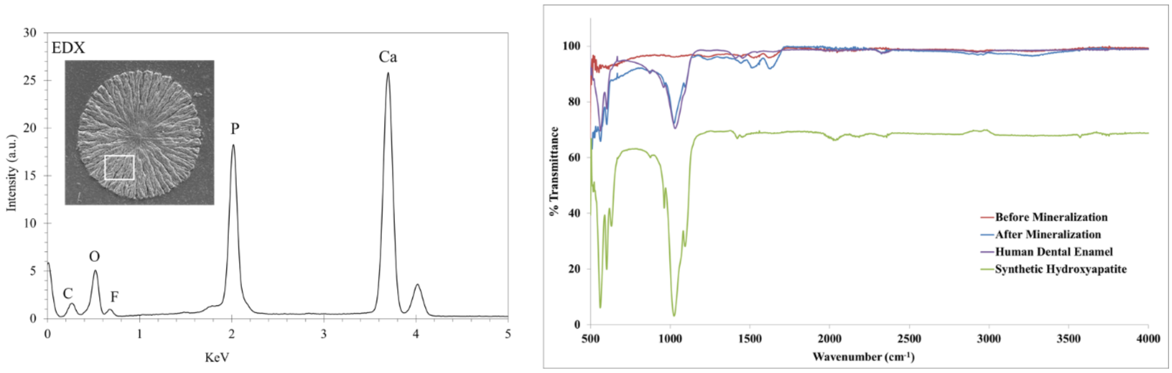

Supplementary Figure 7. EDX mapping (left) of the structures showing the presence of calcium, phosphorus, and fluoride with atomic ratios similar to stoichiometric apatite crystals and dental hard tissues. Fourier transform infra-red (FTIR) spectroscopy analysis (right), which revealed spectra exhibiting amide peaks before undergoing mineralization (corresponding to the statherin-rich ELR material), while after mineralization they exhibited hydroxyl-free apatite peaks (OH liberation peak at 630 cm^{-1} is absent in comparison to pure apatite where this peak is evident), as confirmed by MAS-NMR.

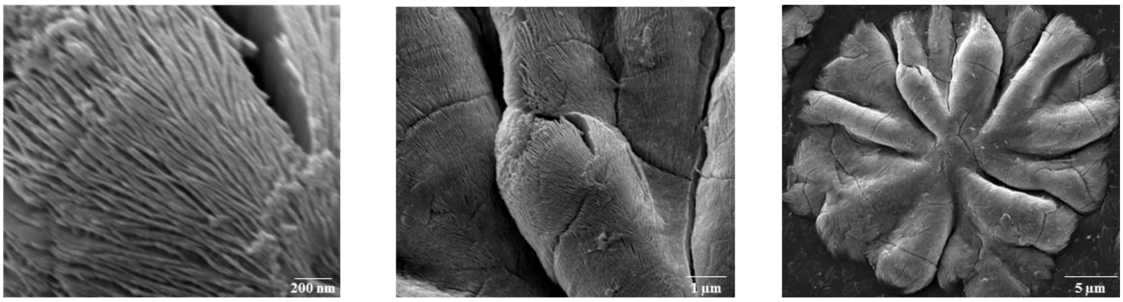

Supplementary Figure 8. SEM images of RGDS-ELR membranes, when mineralized without the use of fluoride, showing a similar hierarchical organisation with those formed with fluorine. However, the morphology of nanocrystals changed to elongated plate-like due to the different chemistry.

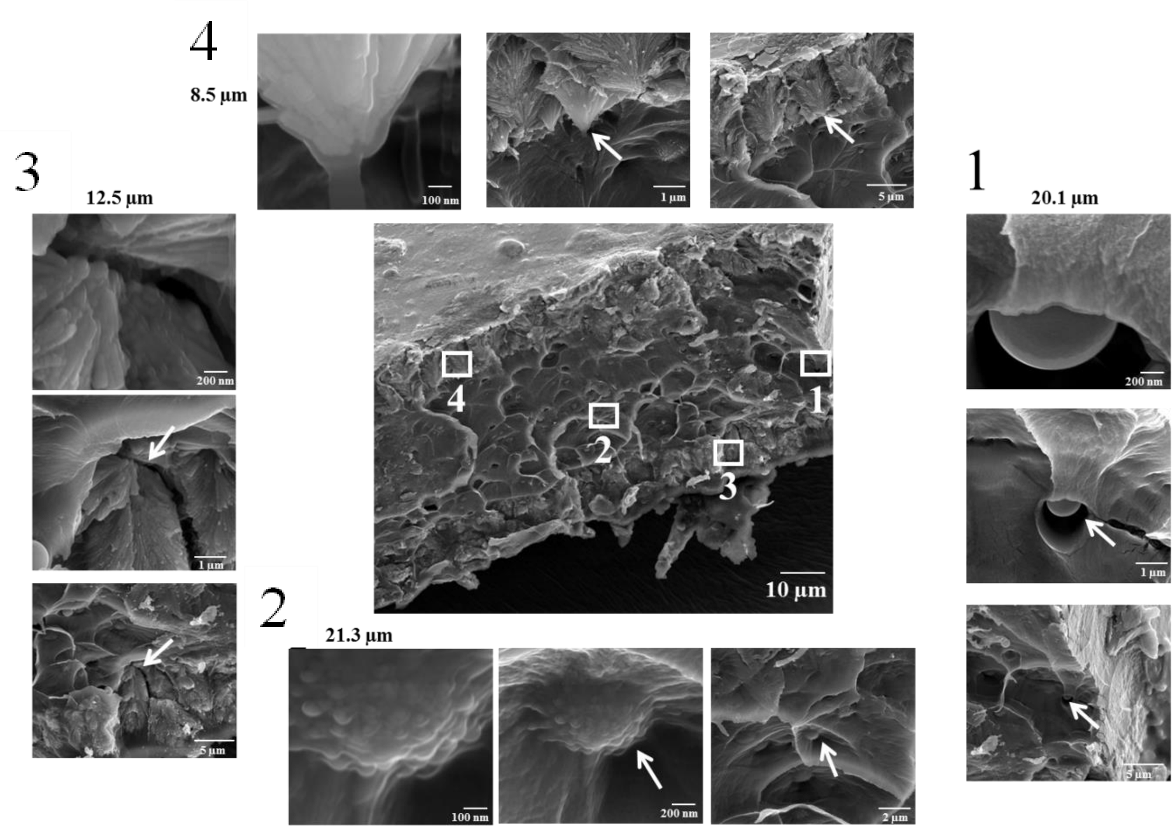

Supplementary Figure 9. SEM images showing the nucleation and crystal growth within the bulk of the organic matrix revealed different structures. Round structures exhibiting a dense pattern of regular granular regions were observed deep within the bulk of the membrane while core structures made from nanocrystals oriented at 78±6° with respect to the membrane surface, were observed nearer the surface. It is possible that these two types of structures represent different stages of development of the mineralized cores, suggesting the presence of an ionic gradient across the cross-section of the organic matrix.

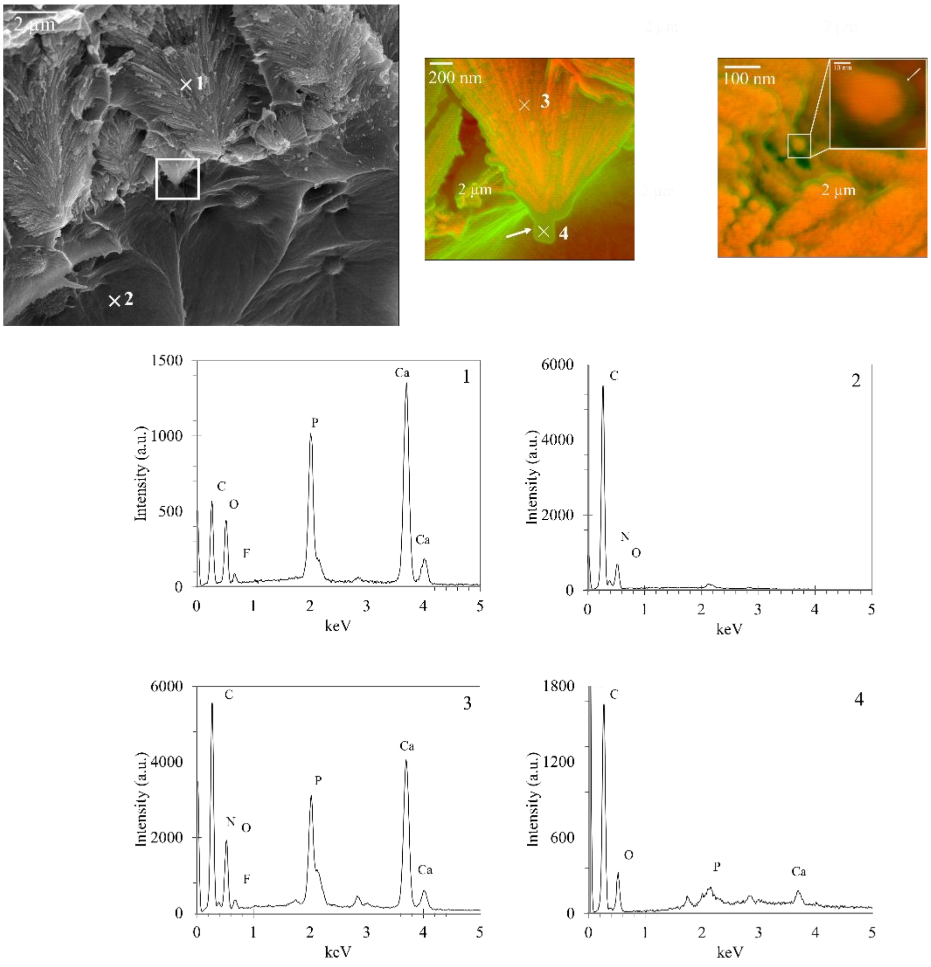

Supplementary Figure 10. Closer examination using DDC-SEM and EDX spectral mapping of both of these structures revealed a thin less dense material (green) surrounding a denser (orange) material. The less dense material was found to be rich in carbon, oxygen, and nitrogen, which is commonly found in organic material. In contrast, the denser material exhibited abundance of calcium and phosphorus, which reflects its inorganic nature.

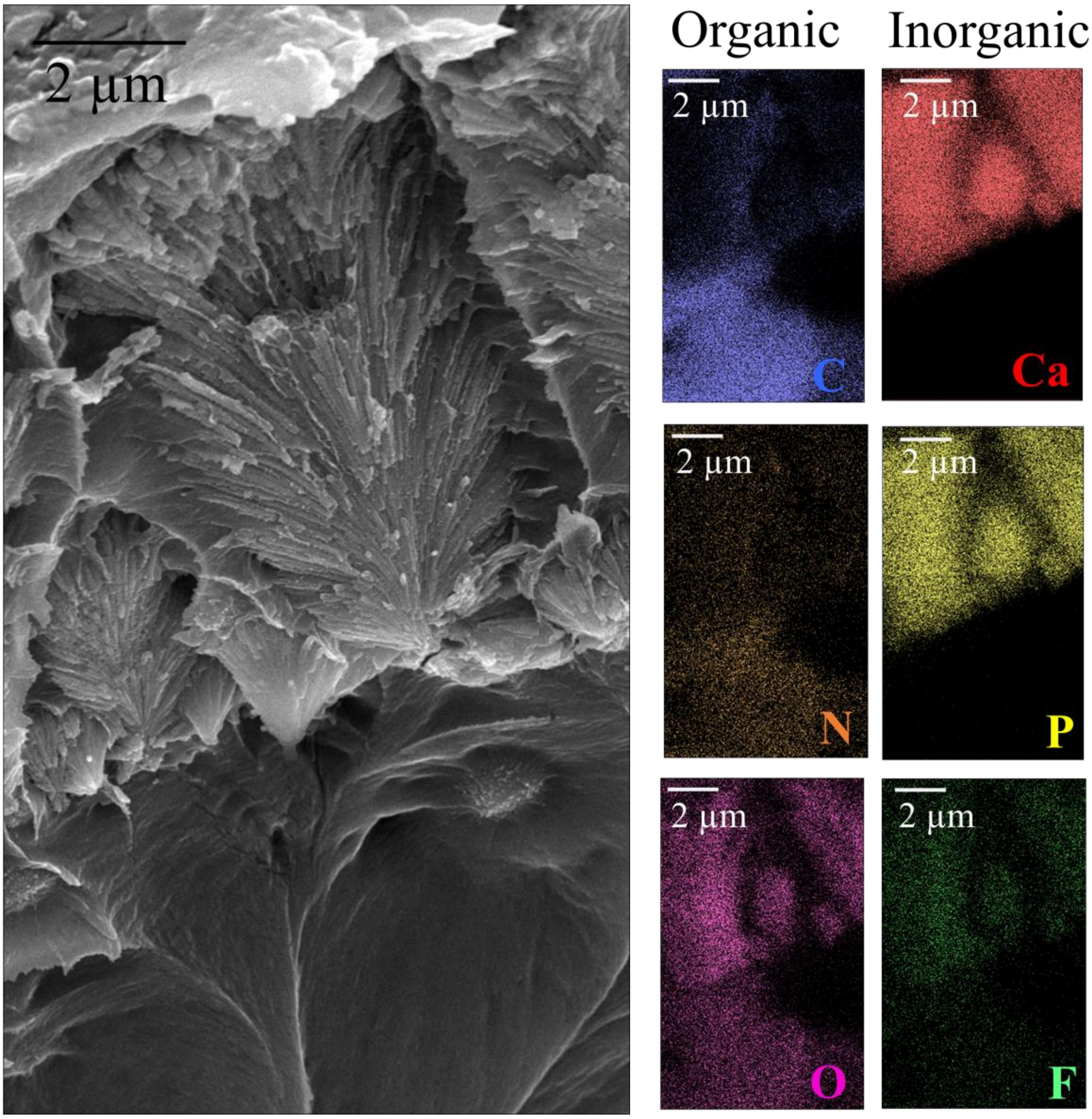

Supplementary Figure 11. Cross-section of the RGDS-ELR membrane (left) after 8 days of mineralization showing the two different morphologies found within the bulk; prismatic structure (e) and spherulite-like structures found within the membrane and suggesting the presence of ionic gradient. EDX mapping (right) at the same area of the SEM image, near the surface of the membrane, calcium, phosphorus, fluoride, and oxygen are abundant representing the prismatic crystalline structures. Away from the surface, carbon, nitrogen, and oxygen elements exhibit higher signals reflecting the organic nature of the ELR spherulites. This elemental distribution gives an indication of the presence of both organic and inorganic materials.

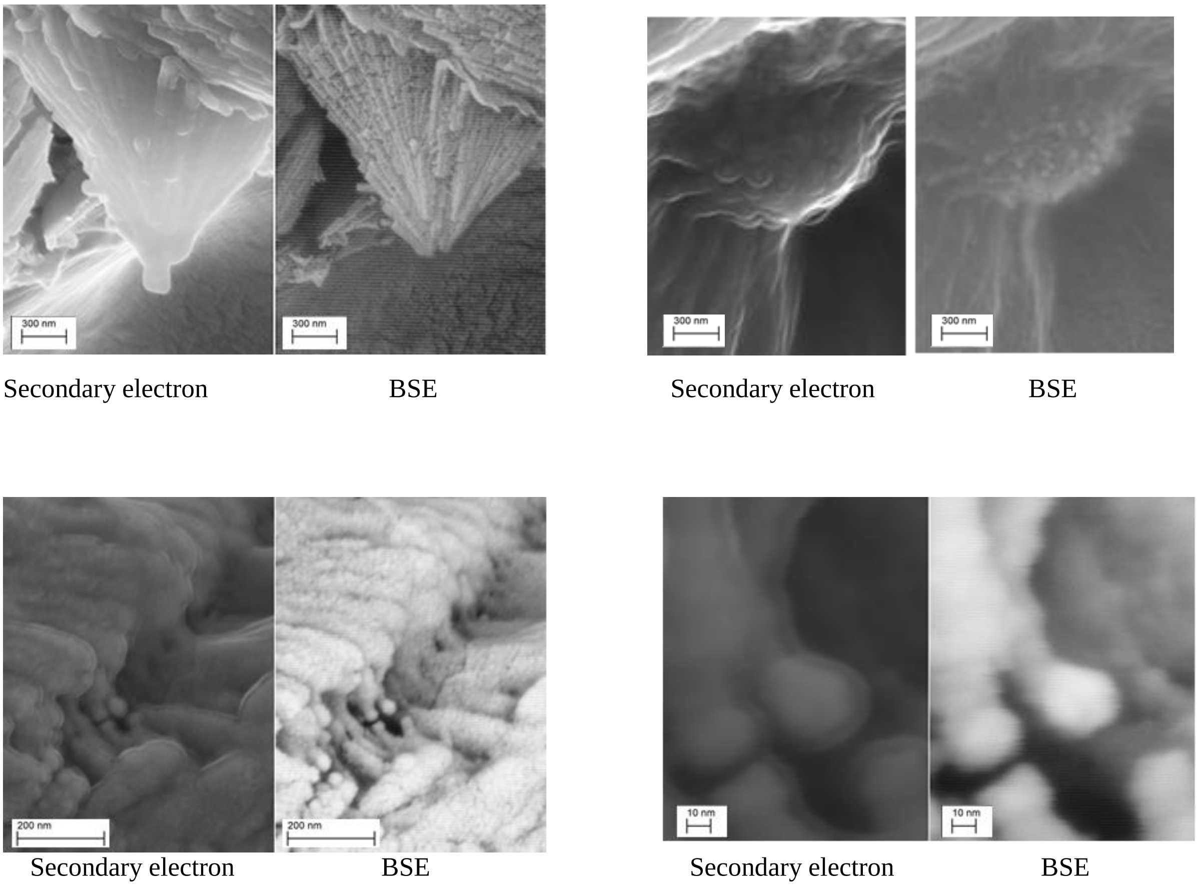

Supplementary Figure 12. SEM and BSE images taken simultaneously from same area to allow density-dependent analyses (DDC-SEM). BSE images show clearly the disappearance of the thin coating around the crystals giving an indication of the presence of the less dense material surrounding the crystals. The less dense material is confirmed in (Supplementary Figure 10) to be rich in carbon, nitrogen and oxygen, giving indication of its organic nature.

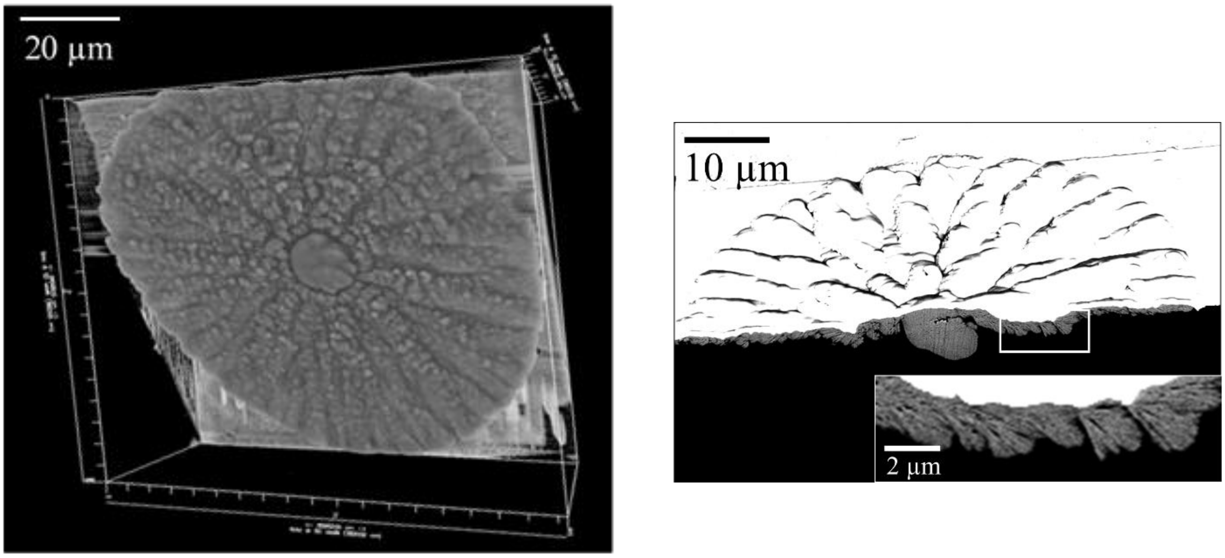

Supplementary Figure 13. 3D reconstruction of FIB-SEM imaging. The angle of visualization is modified in order to be able look at the structures from within the membrane, which also clearly shows the core structure at the centre (left). Scanning electron microscopy (SEM) using the backscattered electron mode (BSE) and focused ion beam (FIB) revealed that the mineralized structures exhibit a mineralized core deep within the membrane made from similar elongated and aligned nanocrystals (right).

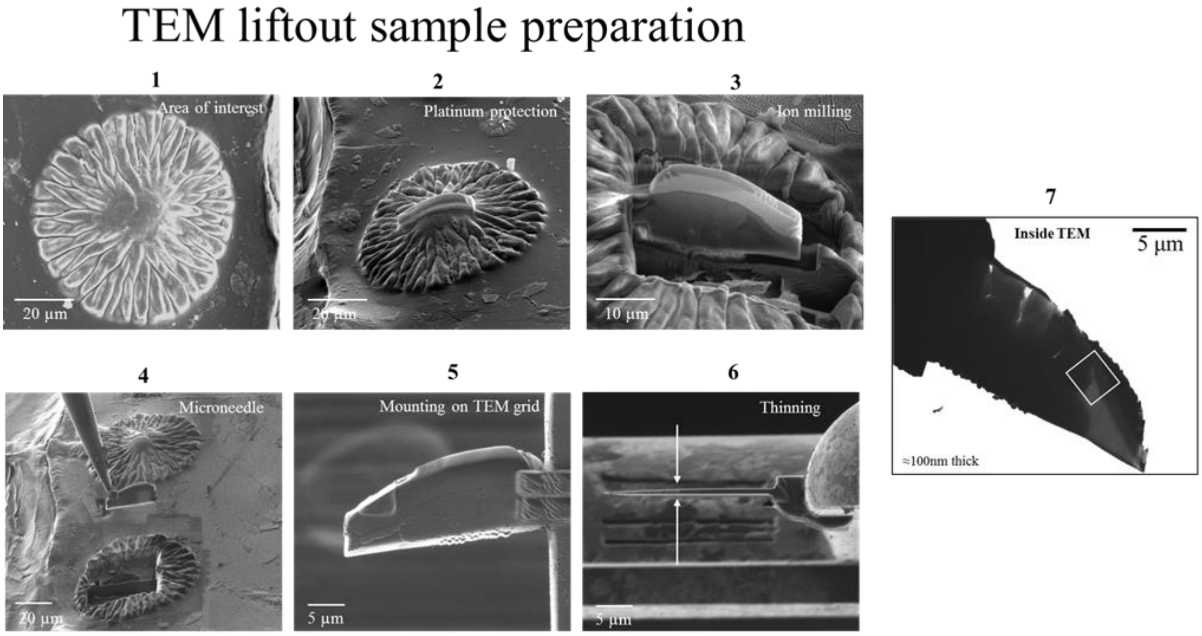

Supplementary Figure 14. Series of SEM images (1-6) showing the procedures followed to prepare the samples for the TEM liftout using FIB. The lamellae (7) is thinned down in order to be transmit electrons through using the TEM for structural and crystallographic information.

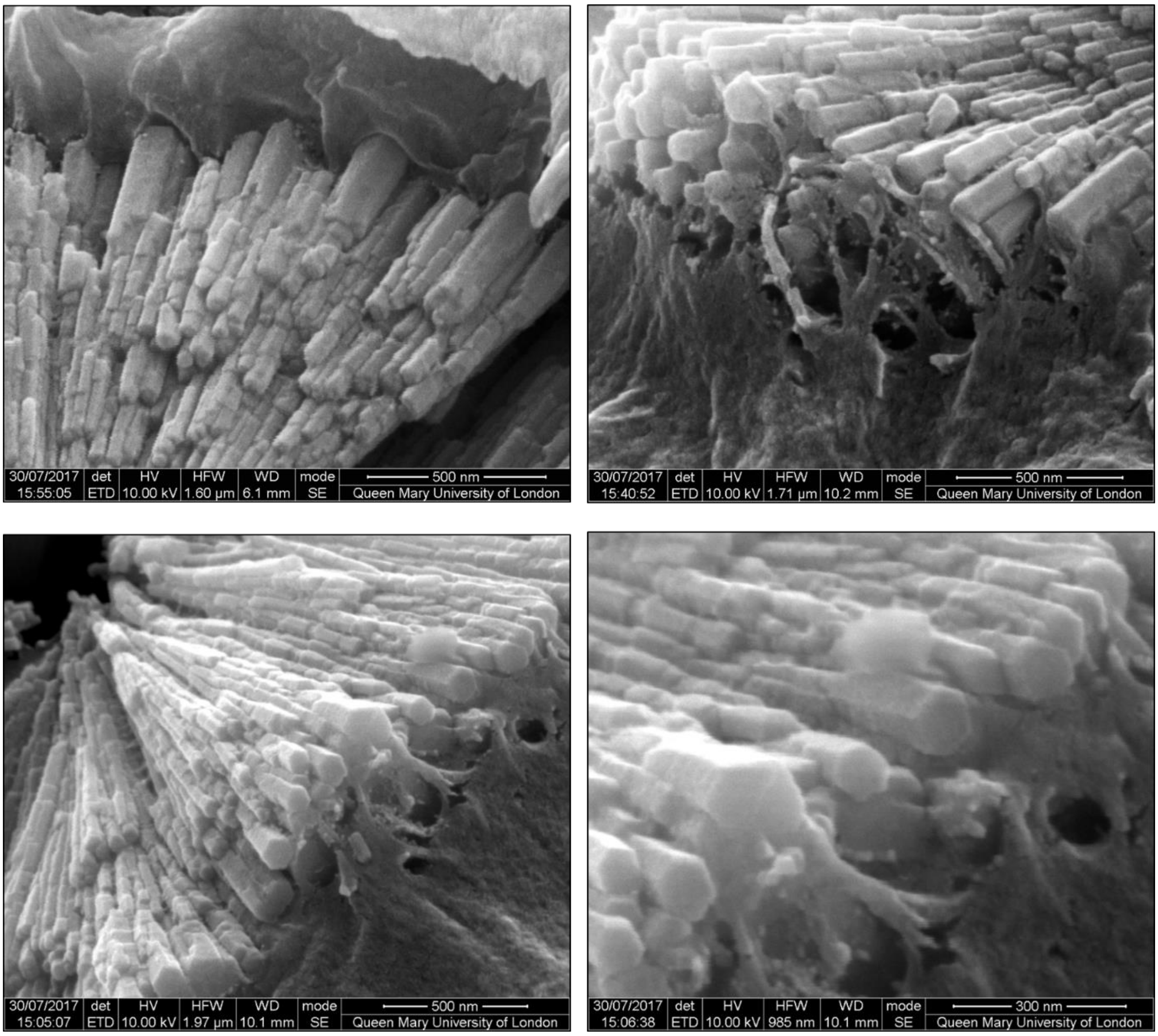

Supplementary Figure 15. SEM images further confirm the intimate organic-inorganic relationship; first by the presence of embedded nanocrystals within the organic material and second by the flat geometry at the end of the nanocrystals, which further offer evidence that the ELR not only interacts at the basal plane of the fluorapatite crystals but also provides an optimum environment for the growth front of the crystals within the matrix.

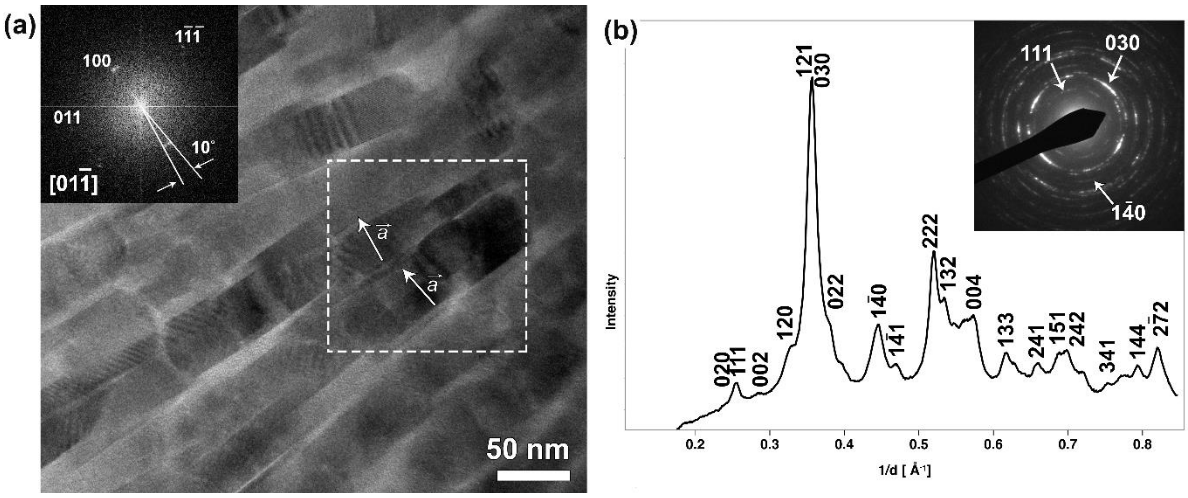

Supplementary Figure 16. HRTEM image of the crystal's co-alignment. The dotted line marks the area from which the Fast Fourier transform (shown in the inset, left) has been taken. Arrows denote the directions of the *a*-axes of the crystals; (b) Radial average of the SAED pattern (shown in the inset, right), taken from the sample area marked in (a). The Fast Fourier transform, SAED pattern and Radial profile have been indexed in the fluorapatite crystal structure (sp.gr. $P6_3/m$).

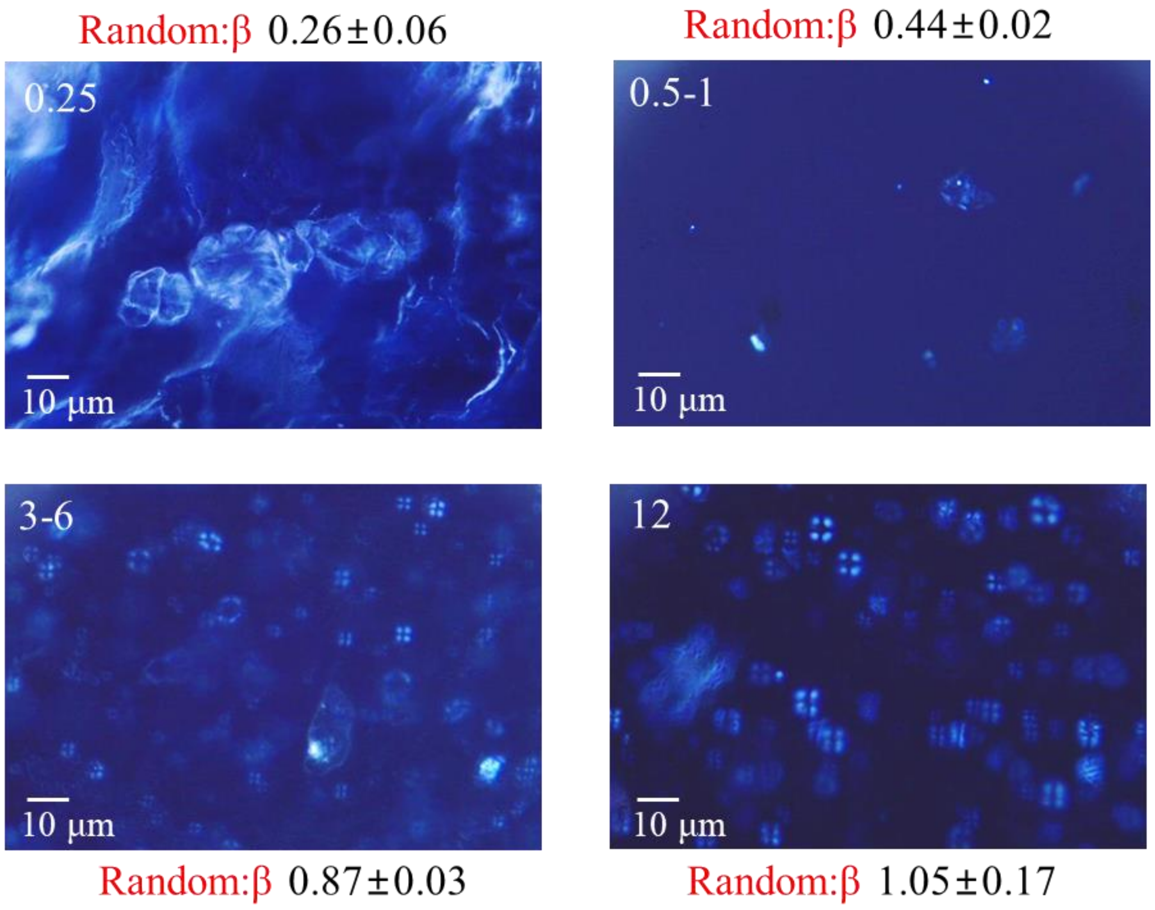

Supplementary Figure 17. Using the drying/cross-linking approach, we found that it was possible to control the formation of ELR spherulites. When the ratio of disordered (random coil) to ordered (β-sheet) secondary structure (random : β ratio) was kept below 0.26 ± 0.06, no spherulites were formed. However, by gradually increasing the level of random coil (random : β ratios of 0.44 ± 0.02, 0.87 ± 0.03, and 1.05 ± 0.17), we were able to control the formation and number of ELR spherulites (0.17, 3.24, and up to 5.38 spherulites/mm^2, respectively), demonstrating that ELR disorder-order interplay can be used to generate organic matrices with supramolecular control.

ELR crosslinks	α-helix	β-turn	Random coil	β-sheet	Random : β
0.25	11.29	10.34	18.51	59.83	0.31
	11.44	12.32	11.82	64.38	0.18
	11.71	10.72	17.94	59.61	0.3
Ave	11.48	11.13	16.09	61.27	0.26
SD	0.17	0.86	3.03	2.20	0.06
0.5-1	7.06	8.77	26.39	57.74	0.46
	6.95	8.18	26.71	58.12	0.46
	7.03	9	24.44	59.5	0.41
Ave	7.01	8.65	25.85	58.45	0.44
SD	0.05	0.35	1	0.76	0.02
3-6	7.5	7.7	38.31	45.46	0.84
	4.66	8.58	40.18	46.55	0.86
	4.66	8.58	41.27	45.47	0.91
Ave	5.61	8.29	39.92	45.83	0.87
SD	1.34	0.41	1.22	0.51	0.03
12	8.76	5.69	44.83	40.7	1.1
	8.89	6.41	37.98	46.7	0.81
	9.9	3.55	47.57	38.96	1.22
Ave	9.18	5.22	43.46	42.12	1.05
SD	0.51	1.21	4.03	3.32	0.17

Supplementary Table 2. We discovered that by systematically modifying the amount of cross-linker, it was possible to modulate the levels of ELR ordered β-sheet structure and disordered random coil, while maintaining β-turn and α-helix conformations nearly constant. In other words, tuning the amount of ELR cross-links during solvent evaporation enabled controlled access to different ELR disorder/order ratios within the resulting membrane.

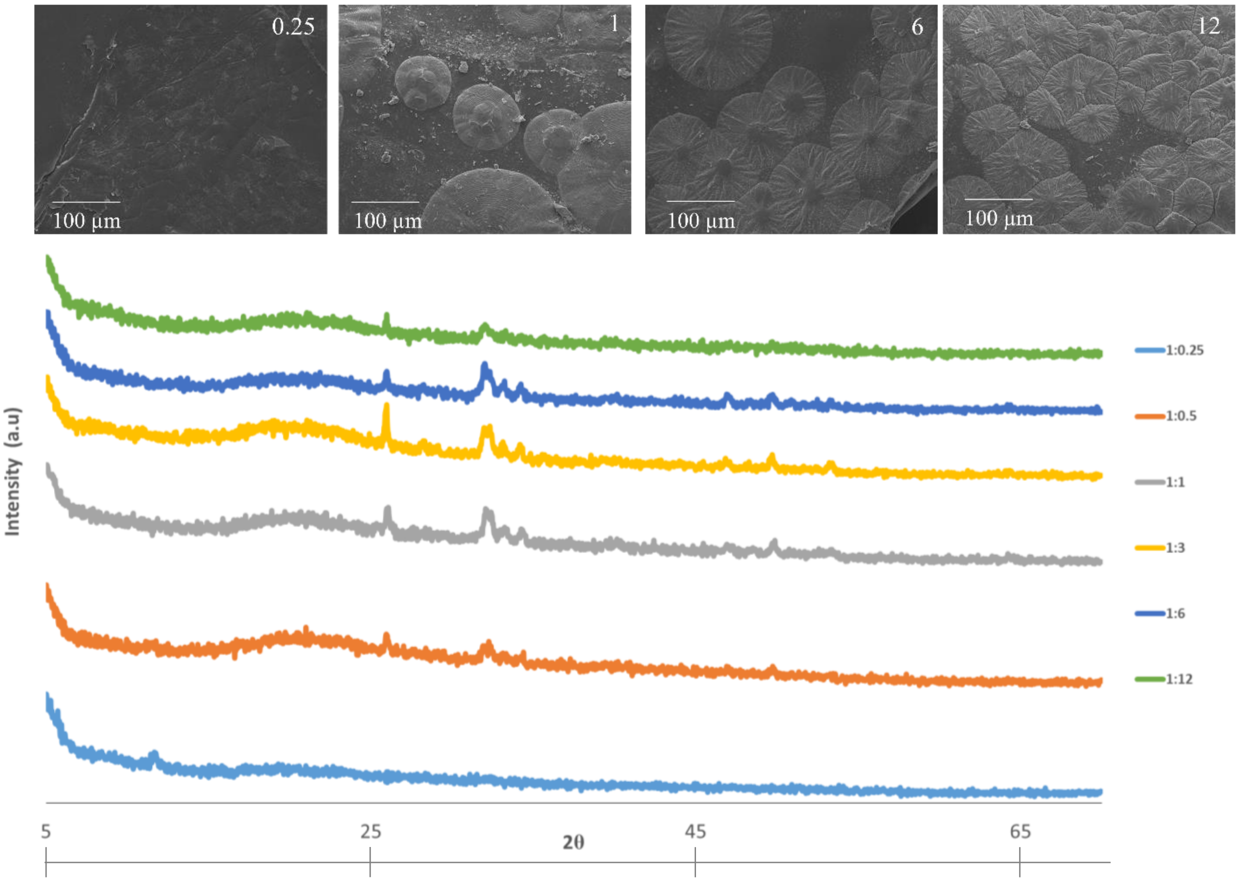

Supplementary Figure 18. The apatite chemical composition of the different organised structures, remains the same as evidenced by XRD at different cross-linking ratios. Note the increase in the number of the structures at high cross-linking.

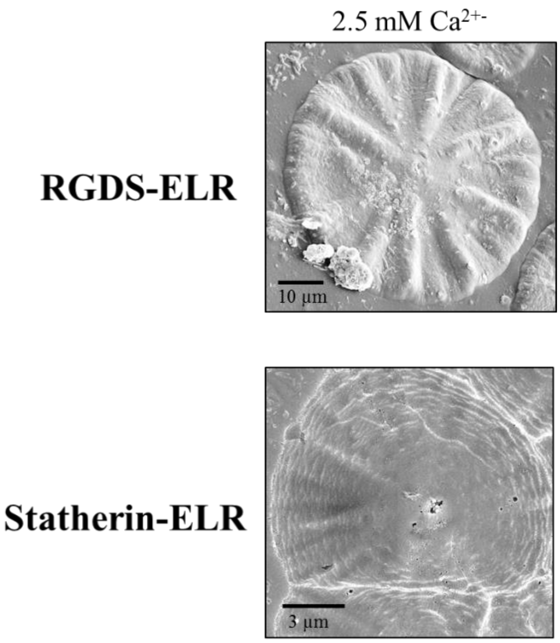

Supplementary Figure 19. SEM of images of mineralized membranes at initial ionic concentrations ranging from 2.5 mM of Ca^{2+}, 1.6 mM of PO_4^{3-}, and 0.5 mM of F^-. This demonstrates that the hierarchical structures form in physiological ionic strength similar to those found in human saliva.

Crosslinking	0.25	0.5-1	3-6	12
Computer modelling	X	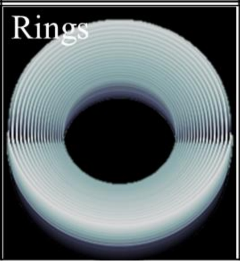	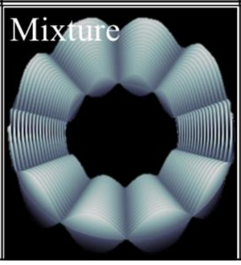	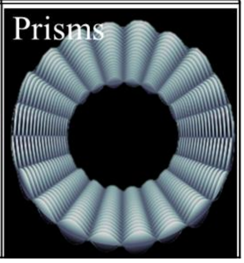

Supplementary Figure 20. To investigate the emergence of the micro-scale prismatic structures, we used a model based on the buckling of a supported elastic thin circular annulus[2]. Geometrically, the assumption is that a mineralized structure with the spherulite-like shape is a thin circular annulus with thickness t, inner radius r_o, and outer radius r_1, supported by the ELR matrix. Mechanically, since the thickness of the mineralized structure is smaller than its inner radius $t \ll r_0$ (Supplementary Movies 1-2), the mineralized structure exhibits low bending stiffness and therefore is unable to withstand compressive stress in the principal directions. For this reason, the mineralized structure would respond with a circumferential out-of-plane deflection when radial compressive loads, as a result of the radially outward nanocrystal growth, are applied to either the inner or outer edge. The assumption is that, the stiffer the ELR matrix the higher the number of elastic supports that can be provided to the mineralized structure. This assumption is based on our observation that the stiffer ELR matrix comprises mineralized structures with more separated nanocrystals than those on the softer ELR matrix, which would lead to higher integration between the ELR matrix (support) and the mineralized structure (annulus). In this scenario, if a compressive radial load $\sigma_0 > 0$ is applied uniformly along the inner radius, membranes with lower elastic supports will wrinkle with a circular symmetry (the ring morphology) because of the inability to withstand radial compression. On the other hand, when more elastic supports are present, the wrinkles will possess, instead, a radial symmetry (the prism morphology), to accommodate the radial lines of zero displacements. The partial differential

equation describing the wrinkling $w(r,\theta)$, where r,θ are the polar coordinates, is the Kirchhoff equation in dimensionless form

$$\nabla^4 w + \beta \frac{1}{r^2}\left[\frac{\partial^2 w}{\partial r^2} - \frac{1}{r}\frac{\partial w}{\partial r} - \frac{1}{r^2}\frac{\partial^2 w}{\partial \theta^2}\right] = 0$$

with β being the critical load parameter

$$\beta = \frac{t\,\sigma_0\,r_0^2}{D}$$

where D is the bending stiffness

$$D = \frac{Et^3}{12(1-\nu)}$$

with E the Young Modulus of the membrane, and ν the Poisson ratio.

Being $t \ll r_0$, the bending stiffness is very low.

Hence, the critical load beta

$$\beta = 12(1-\nu)\frac{\sigma_0}{E}\left(\frac{r_0}{t}\right)^2$$

The solution of the Kirchhoff equation can be expressed as

$$w(r,\theta) = W(r)\cos(n\theta)$$

where n is the number of radial supports. For n=0, the rings morphology is obtained, while $n > 0$ the prism morphology results instead.

The function $W(r)$ satisfies the equation

$$\frac{d^4W}{dr^4} + \frac{2}{r}\frac{d^3W}{dr^3} - \frac{(1+2n^2-\beta)}{r^2}\frac{\partial^2 W}{\partial r^2} + \frac{(1+2n^2-\beta)}{r^3}\frac{\partial W}{\partial r} - \frac{n^2(4-n^2-\beta)}{r^4}W = 0$$

with boundary conditions of simple support at both edges ($r = 1$ for the inner radius and $r = \frac{r_1}{r_0} = \mu$ for the outer radius)

$$W(1) = W(\mu) = 0$$

$$M(1) = M(\mu) = 0$$

where M is the radial moment

$$M = \frac{d^2W}{dr^2} + \frac{\nu}{r}\frac{\partial W}{\partial r} - \frac{\nu}{r^2}W$$

The general solution of the 4th order ordinary differential equation is

$$W(r) = \sum_{i=1}^{4} A_i\, r^{1+\gamma_i}$$

where A_i are coefficients depending on the boundary conditions and

$$\gamma_i^2 = 1 + n^2 - \frac{1}{2}\beta \pm \left(4n^2 - 2\beta n^2 + \frac{1}{4}\beta^2\right)^{1/2}$$

The critical load β is obtained by solving the nonlinear algebraic equation resulting by setting the following determinant to zero:

$$\det \mathbf{A}(\beta) = 0$$

with

$$\mathbf{A}(\beta) = \begin{bmatrix} f_i(1) \\ \frac{d^2 f_i(1)}{dr^2} + \nu\frac{\partial f_i(1)}{\partial r} - \nu f_i(1) \\ f_i(\mu) \\ \frac{d^2 f_i(\mu)}{dr^2} + \frac{\nu}{\mu}\frac{\partial f_i(\mu)}{\partial r} - \frac{\nu}{\mu^2} f_i(\mu) \end{bmatrix}$$

where $f_i = r^{1+\gamma_i}$

Once n is set, it is possible to solve the nonlinear equation and obtain β. Once β is known, it is possible to obtain the coefficients A_i and plot the wrinkling solution. For the results in Fig. 3, we employed $\mu = 2, \nu = 0.3$ and three values of $n = 0, 10, 20$. These three values were chosen based on the number of prisms observed in the SEM images. The eigenvalues analysis led to similar values of $\beta = 25165$, meaning that, the three observed morphologies can exist at approximately the same critical load, with the discriminating factor being the number n of radial line supports.

Cross-linker to Lysine ratio	Equilibrium radius squared (r^2) (mm)	Characteristic time (τ) of swelling (minutes)	Diffusion coefficient (r^2/τ) (mm^2/minute)
0.5	2994.2784	50	59.885568
1	2829.1761	120	23.5764675
3	2702.9601	240	11.26233375
6	2642.9881	300	8.809960333
12	2611.21	420	6.217166667

Supplementary Table 3. Table showing the Diffusion coefficients of the membranes at different cross-linking ratios.

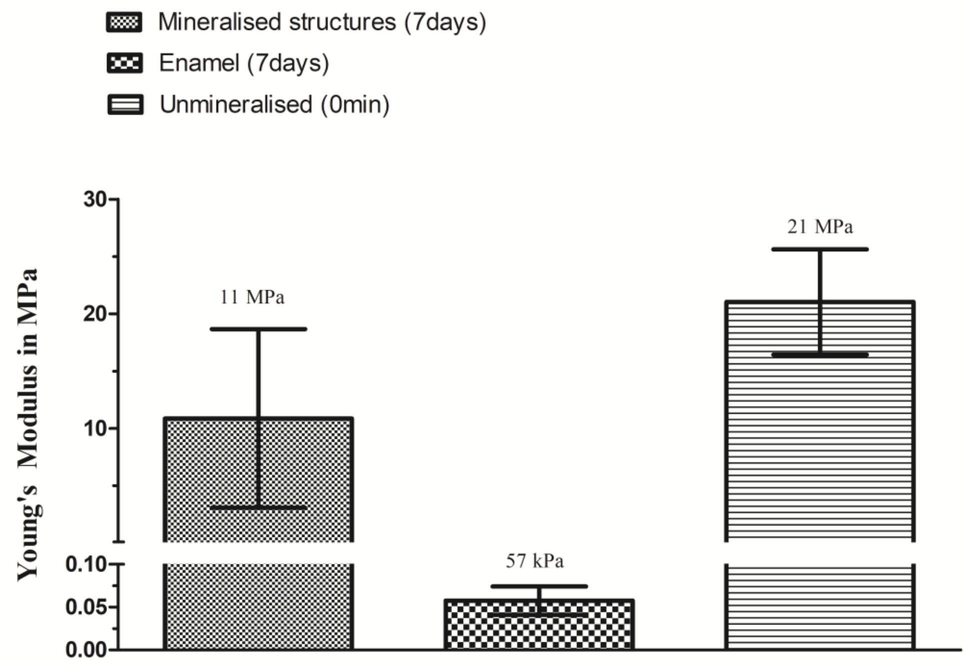

Supplementary Figure 21. As expected, after 7 days in the acid exposure, the inorganic content of the mineralized membranes and dental enamel were disturbed. This result is confirmed by AFM nanoindentation studies where both the mineralized and unmineralized membranes exposed to the acid attack for 7 days exhibited a similar stiffness, suggesting that the organic matrix is maintained. The preservation of the organic matrix could enable a remineralization treatment once the acid attack subsides. However, confirmation of this hypothesis would require further experimentation, which is beyond the scope of the current study. Error bars are represented by standard error of the mean (SEM), n = 9.

Supplementary References

1 Sudarsanan, K., Mackie, P. E. & Young, R. A. Comparison of synthetic and mineral fluorapatite, Ca5 (PO4)3F, in crystallographic detail. *Materials Research Bulletin* **7**, 1331-1337 (1972).

2 Mansfield, E. H. On the buckling of an annular plate. *Quarterly Journal of Mechanics and Applied Mathematics* **13**, 16-23 (1960).
