# Supplementary material for: Protein disorder–order interplay to guide the growth of hierarchical mineralized structures
Source: Nat Commun. 2018 Jun 1;9:2145. doi: 10.1038/s41467-018-04319-0 (PMC5984621; doi:10.1038/s41467-018-04319-0)
Supplement: Supplementary file 2 — Description of Additional Supplementary Files [file 41467_2018_4319_MOESM2_ESM.docx]

**Description of Additional Supplementary Files**

File Name: Supplementary Movie 1

Description: Movie showing the sequential slicing of the hierarchical mineralized structures using Scanning electron microscopy (SEM) using the backscattered electron mode (BSE) and focused ion beam (FIB). A characteristic core structure made from elongated and aligned nanocrystals can be visualized at the center of the hierarchical structures.

File Name: Supplementary Movie 2

Description: Movie showing the sequential slicing of the hierarchical mineralized structures using Scanning electron microscopy (SEM) using the backscattered electron mode (BSE) and focused ion beam (FIB). A characteristic core structure made from elongated and aligned nanocrystals can be visualized at the center of the hierarchical structures.
